# Immunoinformatics and analysis of antigen distribution of *Ureaplasma diversum* strains isolated from different Brazilian states

**DOI:** 10.1186/s12917-020-02602-1

**Published:** 2020-10-07

**Authors:** Manoel Neres Santos Junior, Ronaldo Silva Santos, Wanderson Souza Neves, Janaina Marinho Fernandes, Bruna Carolina de Brito Guimarães, Maysa Santos Barbosa, Lucas Santana Coelho Silva, Camila Pacheco Gomes, Izadora Souza Rezende, Caline Novaes Teixeira Oliveira, Nayara Silva de Macêdo Neres, Guilherme Barreto Campos, Bruno Lopes Bastos, Jorge Timenetsky, Lucas Miranda Marques

**Affiliations:** 1 Department of Biointeraction, Multidisciplinary Institute of Health, Universidade Federal da Bahia, Rua Hormindo Barros, 58 - Quadra 17 - Lote 58, Bairro Candeias - CEP: 45.029-094, Vitória da Conquista, BA Brazil; 2 Department of Microbiology, State University of Santa Cruz (UESC), Ilhéus, Brazil; 3 Department of Microbiology, Institute of Biomedical Science, University of São Paulo, São Paulo, Brazil

**Keywords:** *Ureaplasma diversum*, Immunoinformatics, Lipoproteins, Prediction

## Abstract

**Background:**

*Ureaplasma diversum* has numerous virulence factors that contribute to pathogenesis in cattle, including Lipid-associated membrane proteins (LAMPs). Therefore, the objectives of this study were to evaluate in silico important characteristics for immunobiological applications and for heterologous expression of 36 LAMPs of *U. diversum* (UdLAMPs) and, also, to verify by conventional PCR the distribution of these antigens in strains of Brazilian states (Bahia, Minas Gerais, São Paulo, and Mato Grosso do Sul). The Manatee database was used to obtain the gene and peptide sequences of the antigens. Similarity and identity studies were performed using BLASTp and direct antigenicity was evaluated by the VaxiJen v2.0 server. Epitope prediction for B lymphocytes was performed on the BepiPred v2.0 and CBTOPE v1.0 servers. NetBoLApan v1.0 was used to predict CD8^+^ T lymphocyte epitopes. Subcellular location and presence of transmembrane regions were verified by the software PSORTb v3.0.2 and TMHMM v2.2 respectively. SignalP v5.0, SecretomeP v2.0, and DOLOP servers were used to predict the extracellular excretion signal. Physico-chemical properties were evaluated by the web-software ProtParam, Solpro, and Protein-sol**.**

**Results:**

In silico analysis revealed that many UdLAMPs have desirable properties for immunobiological applications and heterologous expression. The proteins gudiv_61, gudiv_103, gudiv_517, and gudiv_681 were most promising. Strains from the 4 states were PCR positive for antigens predicted with immunogenic and/or with good characteristics for expression in a heterologous system.

**Conclusion:**

These works contribute to a better understanding of the immunobiological properties of the UdLAMPs and provide a profile of the distribution of these antigens in different Brazilian states.

## Background

*U. diversum*, a member of the *Mollicutes* class, is a bovine pathogen related to reproductive disorders [1]*.* This agent presents the following outstanding characteristics: the production of ammonia, through urea hydrolysis, and the absence of a cell wall [2]*.* Although *U. diversum* infection is not conditioned by the presence of clinical symptoms, it can colonize the respiratory and genital/reproductive systems of cattle, generating severe inflammatory conditions often culminating in abortion [3]. It is considered an opportunistic pathogen found in the mucosa and secretions of the vulva, vagina, and udder of cows and secretion of the respiratory tract of calves [1].

Milk production in cows and spermatogenesis in bulls are also affected. *U. diversum* produces mastitis along with visible changes in the milk and udder [4]. In bulls, it causes seminal vesiculitis, balanoposthitis, epididymitis, and morphological and functional changes in sperm. Thus, *U. diversum* colonizes different regions of the reproductive system leading to active semen contamination [5]. *Infection of semen for artificial insemination and* in vitro *fertilization results in serious obstacles to modern bovine reproduction techniques* [6].

In addition to urease, *U. diversum* has sophisticated virulence mechanisms, including LAMPs, a mixture of mycoplasmic lipoproteins expressed on the cell surface that interact directly with host cells. These antigens are considered the main molecular agents associated with pathogens in several *Mollicutes* species and play an important role in host pathogenicity and immunomodulation [7]. In addition to lipoproteins, in the bovine ureaplasma genome, our research group identified genes encoding the multiple band antigen (MBA), which contain multiple series repetitions in the C-terminal region, as well as the gene for hemolysin and for the Mycoplasma Ig binding protein (MIB) and Mycoplasma Ig protease (MIP) –MIB-MIP system-, which acts by binding and cleaving the IgG heavy chain [2, 8].

The genomic sequencing of a species offers researchers new possibilities for research. Rapid analysis of all or part of the genome allows the construction of primers and screening of genes coding for virulence factors in the most diverse bacterial strains. The use of immunoinformatics tools allows screening with a high level of reliability of the physico-chemical and immunological properties of these molecules with low cost and reliable results [9]. The use of recombinant DNA technology can, through expression in a heterologous system, allow the analysis of virulence factors alone. Therefore, the objective of this work was to evaluate antigens of *U. diversum* regarding immunobiological properties and desirable characteristics for expression in a heterologous system, as well as to evaluate the distribution of these antigens in isolates from different regions of Brazil.

## Results

### *U. diversum* antigens have low similarity with bovine proteome proteins

BLASTp analyses of the 36 UdLAMPs with bovine proteomes revealed that the maximum similarity occurred between the lipoprotein gudiv_159 and the Tinken-1 protein from *Bos taurus taurus* (29%). *Bos taurus indicus* had a similarity detected only for gudiv_517 (10%). The hybrid showed no significant similarity to any protein (Table 1).

**Table 1:** Analysis of similarity between sequences of 36 UdLAMPs and proteomes of bovine subspecies (*Bos taurus taurus*, *Bos taurus indicus* and the *hybrid Bos taurus x Bos indicus*) performed using the BLASTp tool

UdLAMPs	Similarity with different bovine proteomes^b^		
	*Bos taurus taurus*	*Bos taurus indicus*	Hybrid^a^
**gudiv_61**	–	–	–
**gudiv_66**	–	–	–
**gudiv_85**	–	–	–
**gudiv_91**	11%	–	–
**gudiv_93**	7%^a^	–	–
**gudiv_103**	–	–	–
**gudiv_159**	29%	–	–
**gudiv_162**	–	–	–
**gudiv_164**	–	–	–
**gudiv_171**	9%	–	–
**gudiv_179**	–	–	–
**gudiv_180**	4%	–	–
**gudiv_228**	–	–	–
**gudiv_262**	–	–	–
**gudiv_287**	10%	–	–
**gudiv_331**	–	–	–
**gudiv_357**	–	–	–
**gudiv_388**	–	–	–
**gudiv_398**	–	–	–
**gudiv_402**	–	–	–
**gudiv_410**	–	–	–
**gudiv_412**	–	–	–
**gudiv_427**	–	–	–
**gudiv_442**	–	–	–
**gudiv_457**	17%	–	–
**gudiv_458**	–	–	–
**gudiv_499**	–	–	–
**gudiv_517**	–	10%	–
**udiv_546**	–	–	–
**gudiv_560**	–	–	–
**gudiv_633**	8%	–	–
**gudiv_635**	–	–	–
**gudiv_663**	–	–	–
**gudiv_680**	–	–	–
**gudiv_681**	–	–	–
**gudiv_759**	–	–	–

^a^hybrid: Bos taurus taurus x Bos taurus indicus

^b^Only the maximum similarity found

^−^ Similarity not significant by BLASTp

### In silico analysis showed that *U. diversum* antigens have epitopes for B and T lymphocytes

Conformational and linear B cell epitopes were evaluated for the number of regions and the total percentage of amino acids in epitope regions. All proteins showed conformational epitopes for B lymphocytes. The most significant B cell epitopes are listed in Additional Table 1. The number of antigenic regions ranged from 2 in gudiv_388 to 124 in gudiv_398. The proteins with the lowest and highest percentage of amino acids in antigenic regions were gudiv_164 (4.3%) and gudiv_66 (39.9%) respectively (Table 2). Except for 10 proteins (gudiv_546, gudiv_457, gudiv_427, gudiv_442, gudiv_388, gudiv_357, gudiv_331, gudiv_228, gudiv_171 and gudiv_159), all the others have a number of predicted regions greater than or equal to the values for surface protein 5 (Msp5) from *Anaplasma marginale* (Table 3). In the prediction of linear epitopes the number of antigenic regions varied from 1 in gudiv_159 to 84 in gudiv_398. The protein with the highest percentage of amino acids in antigenic regions was gudivi_179 (90.4%). Thirty proteins had number of antigenic regions greater than or equal to Msp5. Eighteen of the 36 UdLAMPs were predicted to be antigenic (score greater than or equal to 0.5 on the VaxiJen server).

**Table 2:** Prediction of antigenicity and discontinuous and continuous B lymphocyte epitopes of each UdLAMP by the predictors CBTOPE v1.0, BepiPred v2.0, and VaxiJen v2. 0

UdLAMPs	conformational epitopes (CBTOPE)		linear epitopes (BepiPred)		antigenicity (VaxiJen)
	number of predicted regions	% of aa in predicted regions	number of predicted regions	% of aa in predicted regions	
**gudiv_61**	33	23.0	9	61.6	0.54
**gudiv_66**	34	39.9	15	56	0.45
**gudiv_85**	42	26.3	15	65.5	0.91
**gudiv_91**	19	16.7	11	31	0.43
**gudiv_93**	41	18.2	8	88.5	0.71
**gudiv_103**	20	25.1	6	68.2	0.51
**gudiv_159**	10	20.2	1	83.1	1.13
**gudiv_162**	69	37.0	19	73	0.48
**gudiv_164**	58	4.3	7	64.6	0.55
**gudiv_171**	17	16.6	14	57.6	0.31
**gudiv_179**	33	31.4	4	90.4	1.1
**gudiv_180**	68	27.3	25	66.5	0.54
**gudiv_228**	10	9.4	11	47.3	0.48
**guduv_262**	21	17.8	4	77.3	0.48
**gudiv_287**	55	30.3	12	73.1	0.51
**gudiv_331**	4	6.1	2	62.6	1.23
**gudiv_357**	15	13.7	10	36.7	0.56
**gudiv_388**	2	8.2	3	58.8	0.45
**gudiv_398**	124	19.2	84	55.9	0.41
**gudiv_402**	29	20.7	13	62.2	0.77
**gudiv_410**	34	19.6	18	61	0.48
**gudiv_412**	36	15.7	16	61.6	0.5
**gudiv_427**	11	21.4	6	61	0.37
**gudiv_442**	13	14.1	9	68.9	0.45
**gudiv_457**	13	18.6	9	39.8	0.34
**gudiv_458**	36	26.9	20	110	0.49
**gudiv_499**	23	20.1	15	59.1	0.53
**gudiv_517**	19	21.0	5	63.3	0.58
**gudiv_546**	13	19.0	4	59.2	0.29
**gudiv_560**	32	17.2	17	59.2	0.48
**gudiv_633**	24	14.0	17	63.2	0.48
**gudiv_635**	34	23.9	21	59.3	0.41
**gudiv_663**	51	26.9	21	57.5	0.58
**guduv_680**	23	15.7	18	54.1	0.57
**gudiv_681**	25	31.6	6	77.4	0.58
**gudiv_759**	47	32.1	17	65.1	0.47
**Msp5**^a^	**19**	**45.2**	**5**	**43.3**	**0.51**

*Mapping of B lymphocyte epitopes and antigenicity prediction was also performed for the Msp5 ESXA_MYCBO peptide from *A. marginale*

**Table 3:** Prediction of binding of UdLAMPs (peptide windows with 9 amino acids) to different BoLA alleles (MHCI) performed through the NetBoLApan v1.0 server. The total of strong and weak connections is expressed in absolute numbers

UdLAMPs	BoLA-1 *02301	BoLA-3 *00201	BoLA-2 *01201	BoLA-6 *01301	BoLA-3 *00101	BoLA-6* 04101	BoLA- T2C	BoLA – T5
**gudiv_61**	6	5	9	6	5	3	4	3
**gudiv_66**	11	9	8	7	5	5	9	16
**gudiv_85**	0	0	0	0	0	0	0	0
**gudiv_91**	7	7	9	9	11	0	19	10
**gudiv_93**	6	3	12	6	2	10	4	5
**gudiv_103**	4	5	7	3	2	5	3	3
**gudiv_159**	3	0	7	1	0	0	0	0
**gudiv_162**	15	8	24	9	8	13	10	13
**gudiv_164**	4	2	3	10	2	4	9	3
**gudiv_171**	6	8	10	5	5	4	16	11
**gudiv_179**	1	4	12	3	3	3	2	1
**gudiv_180**	9	6	26	9	4	17	13	13
**gudiv_228**	5	5	4	11	2	9	6	8
**gudiv_262**	0	1	3	1	2	4	4	0
**gudiv_287**	10	9	26	10	12	14	19	12
**gudiv_331**	3	0	5	1	0	1	0	0
**gudiv_357**	8	10	3	10	5	7	12	13
**gudiv_388**	2	0	4	1	1	1	1	1
**gudiv_398**	42	33	75	44	28	34	60	57
**gudiv_402**	5	2	8	5	1	6	5	1
**gudiv_410**	6	7	17	8	4	6	12	10
**gudiv_412**	6	8	13	11	4	9	13	11
**gudiv_427**	3	4	3	2	2	3	7	3
**gudiv_442**	5	3	6	6	2	4	9	7
**gudiv_457**	7	1	7	8	3	7	6	7
**gudiv_458**	6	6	20	11	7	3	18	6
**gudiv_499**	6	5	11	6	3	6	6	7
**gudiv_517**	5	5	8	6	2	3	9	6
**gudiv_546**	5	2	6	3	0	3	5	3
**gudiv_560**	8	9	12	10	5	14	15	9
**gudiv_633**	5	5	13	7	0	9	17	11
**gudiv_635**	4	6	16	6	3	9	8	8
**gudiv_663**	8	12	16	11	8	7	16	10
**gudiv_680**	8	5	16	5	5	7	13	6
**gudiv_681**	6	1	6	1	3	2	5	3
**gudiv_759**	6	7	10	8	8	9	16	8
tp2*	**5**	**3**	**6**	**7**	**2**	**5**	**9**	**4**

* Mapping of TCD8+ lymphocyte epitopes was also performed for *Theileria parva* Tp2 antigen

In the prediction for major histocompatibility complex class I (MHCI) ligand, with the exception of gudiv_85 and gudiv_159, all other lipoproteins showed at least one predicted link for 4 of the 8 MHCI alleles bovine lymphocyte antigen (BoLA) studied (Table 3). Epitopes with strong binding in each BoLA allele are listed in Additional Tables 2 and 3. The maximum number of bonds was between the epitopes of the gudiv_398 protein and the BoLA-2 *01201 allele (75 bonds). Only three *U. diversum* antigens (gudiv_85, gudiv_331, and gudiv_388) had fewer connections than the *Theileria parva* 2 antigen (Tp2) in all alleles, of these, gudiv_85 did not show predicted connections in any allele (Table 3).

### Some UdLAMPs have low identity compared to proteomes of other *Mollicutes*

The identity analysis of UdLAMPs with proteomes of other *Mollicutes* (*Mycoplasma bovis, Mycoplasma canadense, Mycoplasma bovigenitalium, Mycoplasma bovirhinis* and *Mycoplasma díspar*) revealed that only 8 proteins (gudiv_103, gudiv_159, gudiv_171, gudiv_228, gudiv_517, gudiv_546, gudiv_680, gudiv_681) did not present a significant identity with the analyzed proteomes. Twenty-four proteins showed an identity greater than 30% (Table 4).

**Table 4:** BLASTp identity analysis of 36 UdLAMPs with proteomes of *M. bovis*, *M. canadense*, *M. bovigenitalium*, *M. bovirhinis* and *M. dispar*

UdLAMPS	***Mycoplasma bovis***	***Mycoplasma canadense***	***Mycoplasma bovigenitalium***	***Mcoplasma bovirhinis***	***Mycoplasma dispar***
**gudiv_061**	29.81%	–	33.42%	–	–
**gudiv_066**	–	–	26.25%	–	–
**gudiv-091**	29.87%	30.03%	24.92%	26.97%	–
**gudiv_085**	–	–	–	31.91%	–
**gudiv_093**	36.43%	30.3%	29.4%	26.97%	28.92%
**gudiv_103**	–	–	–	–	–
**gudiv_159**	–	–	–	–	–
**gudiv_162**	43.95%	44.93%	45.84%	77.42%	35.01%
**gudiv_164**	–	–	76.92%	44.64%	–
**gudiv_171**	–	–	–	–	–
**gudiv_179**	–	34.38%	–	–	–
**gudiv_180**	45.88%	42.65%	41.83%	29.85%	36.36%
**gudiv_228**	–	–	–	–	–
**Gudiv_262**	29.3%	–	–	–	–
**gudiv_287**	39.77%	50%	55.56%	60.26%	39.25%
**gudiv_331**	42.42%	–	–	–	–
**gudiv-357**	31.33%	27.2%	32.93%	33.33%	27.39%
**gudiv_388**	–	–	43.33	–	–
**gudiv_398**	–	51.4%	63.68%	29.76%	26.36%
**gudiv_402**	43.75%	–	–	32.26%	–
**gudiv_410**	54.84%	–	40.82%	–	–
**gudiv_412**	51.61%	36.67%	42.86%	–	–
**gudiv_427**	–	–	–	33.96%	–
**gudiv_442**		28.79%	–	–	–
**gudiv_457**	31.58%	28.32%	29.12%	28.32%	27.59%
**gudiv_458**	–	–	38.03%	50%	–
**gudiv_499**	–	–	37.5%	–	–
**gudiv_517**	–	–	–	–	–
**gudiv_546**	–	–	–	–	
**gudiv_560**	29.11%	–	28.52%	–	–
**gudiv_635**	–	–	47.22%	–	–
**gudiv_663**	54.17%	–	–		33.33%
**gudiv_663**	–	–	46%	34.25%	–
**gudiv_680**	–	–	–	–	–
**gudiv_681**	–	–	–	–	–
**gudiv_759**	30.66%	–	–	27.78%	27.98%

^−^ Identity not significant by BLASTp

### Some UdLAMPs have characteristics for heterologous expression in *Escherichia coli*

Parameters such as molecular weight (PM), instability index, aliphatic index, grand average of hydropathy (GRAVY), and solubility were predicted for *U. diversum* antigens. The protein PM varied between 9.0 and 240.2 (kilodalton) kDa. The proteins with the highest molecular weight were gudiv_398 (240.2 kDa), gudiv_162 (90.5 kDa) and gudiv_180 (88.7 kDa), while with lower molecular weight were gudiv_159, gudiv_85, and gudiv_331 with 13.3; 9.4 and 9.0 kDa (Table 5). The instability rates ranged from 9.16 (gudiv_499) to 67.15 (gudiv_331). In general, when this index is less than 40, proteins are considered stable; therefore, in this study, only 4 proteins (gudiv_93, gudiv_159, gudiv_331, and gudiv_560) were classified as unstable according to the prediction. To assess hydrophobicity, GRAVY was studied, GRAVY positive proteins were only gudiv_91, gudiv_228, gudi_357, and gudivi_546 with values​ of 0.05; 0.12; 0.61 and 0.05, respectively. As for solubility, the proteins gudiv_91, gudiv_171, gudiv_287, gudv_357, gudiv_458, and gudiv_560 were insoluble in both Protein-Sol and SOLpro. Gudiv_91 and gudiv_357 also presented 4 and 7 transmembrane loops, respectively (Table 5). In total, sixteen proteins were predicted to be soluble in the two predictors (Table 5).

**Table 5:** Prediction of physicochemical properties of UdLAMPs. Aliphatic index, PM, GRAVY, and instability index obtained in ProtParam. The solubility was predicted using the server SOLpro and Protein-Sol

UdLAMPS	PROTPARAM				Protein-Sol	SOLpro
	Number of amino acids	PM (kDa)	Instability Index	GRAVY		
**gudiv_061**	404	45.6	24.4	−0.65	Soluble	soluble
**gudiv_066**	393	46.4	38.09	−0.51	Insoluble	soluble
**gudiv_085**	495	52.9	26.61	−0.58	Soluble	soluble
**gudiv_091**	462	52.4	30.08	0.05	Insoluble	insoluble
**gudiv_093**	660	75.8	41.13	−1.18	Soluble	soluble
**gudiv_103**	220	25	26.72	−0.36	Soluble	soluble
**gudiv_159**	124	13.3	44.69	−1.4	Soluble	soluble
**gudiv_162**	799	90.5	33.12	−0.74	Soluble	soluble
**gudiv_164**	280	32.2	34.88	−0.93	Soluble	insoluble
**gudiv_171**	349	41.0	38.73	−0.41	Insoluble	Insoluble
**gudiv_179**	408	43.8	39.13	−1.16	Soluble	Soluble
**gudiv_180**	774	88.7	29.23	−0.74	Soluble	Soluble
**gudiv_228**	224	25.9	29.91	0.12	Soluble	Insoluble
**gudiv_262**	343	39.1	39.42	−1.26	Soluble	Soluble
**gudiv_287**	739	83.7	33.21	−0.58	Insoluble	Insoluble
**gudiv_331**	99	9	67.15	−1.13	–	Soluble
**gudiv_357**	343	39	35.32	0.61	Insoluble	Insoluble
**gudiv_388**	85	9.4	17.65	−0.52	Soluble	Soluble
**gudiv_398**	2052	240.2	28.43	−0.57	Insoluble	–
**gudiv_402**	381	41.9	31.04	−0.72	Soluble	Soluble
**gudiv_410**	454	53.6	27.91	−0.73	Soluble	Insoluble
**gudiv_412**	502	59.2	29.46	−0.68	Soluble	Insoluble
**gudiv_427**	187	22.3	20.89	−0.39	Soluble	Soluble
**gudiv_442**	312	36.4	29.79	−0.36	Soluble	Insoluble
**gudiv_457**	204	24.2	35.81	−0.37	Soluble	Insoluble
**gudiv_458**	520	60.5	30.61	−0.73	Insoluble	Insoluble
**gudiv_499**	318	36	9.19	−0.59	Soluble	Soluble
**gudiv_517**	215	25	27.83	−0.23	Soluble	Soluble
**gudiv_546**	142	16	27.89	0.05	Soluble	Insoluble
**gudiv_560**	522	62.2	41.38	−0.86	Insoluble	Insoluble
**gudiv_663**	573	64.3	23.08	−0.43	Soluble	Insoluble
**gudiv_633**	514	60.2	33.36	−0.65	Soluble	Insoluble
**gudiv_635**	506	59.4	27.59	−0.63	Soluble	Insoluble
**gudiv_680**	434	49.6	38.43	−0.58	Soluble	Insoluble
**gudiv_681**	266	29.8	17.68	−0.6	Soluble	Soluble
**gudiv_759**	533	61.2	35.2	−0.71	Soluble	Soluble

-Results not determined by the predictor

### A considerable number of UdLAMPs have a signal for excretion by the classical and non-classical pathways

The analysis of classical secretion mediated by signal peptide (SP) was performed by SignalP5. This server predicted SP in 29 of the 36 proteins studied. The size of the SPs ranged from 18 to 29 amino acids and all showed a cleavage site for peptidase II (sec / SPII). A cysteine immediately after the cleavage site can be seen in the predicted SPs (Table 6). The DOLOP server, which uses a series of criteria to predict bacterial lipoprotein SPs, including the preferred occurrence of amino acids, ranked 17 of the 29 proteins predicted by SignalP with typical SP lipoprotein carriers. Of the twenty-nine proteins predicted with the presence of SPs by SignalP, twenty-five also showed a prediction of non-classical excretion when submitted to the predictor SecretomeP (non-signal peptide-mediated excretion). In addition, some proteins (gudiv_61, gudiv_93, gudiv_162, gudiv_164, gudiv_179, gudiv_287, gudiv_331, gudiv_388, gudiv_546, gudiv_633, and gudiv_663) not discriminated as having SP for lipoproteins by DOLOP were predicted to be secreted by non-classic pathways (Table 6).

**Table 6:** Prediction of classical secretion of the signal peptide performed in SignalP v5.0 and DOLOP. Prediction of non-classical secretion by SecretomeP v2.0. Subcellular location and number of transmembrane loops predicted in PSORTb v3.0.2 and TMHMM v2.0

UdLAMPs	SignalP			Subcellular Location		
	Peptide signal and clevage site	Type	SecretomeP	TMHMM	PSortb	DOLOP
**gudiv_061**	MRKHKRIALATGLVAGLLATVSVVAVA--CN	Sec/SPII	Yes	1	Unknown	–
**gudiv_066**	MKPNHSAGWLFKSKWFFALTSFSIISVALVS--CH	Sec/SPII	Yes	0	Unknown	Lipoprotein
**gudiv_085**	MKIKKIKYKWMSLAIATTVAAAGISAVLIS--CT	Sec/SPII	Yes	1	Unknown	Lipoprotein
**gudiv_091**	–	–	–	4	Plasma membrane	–
**gudiv_093**	–	Sec/SPII	Yes	0	Unknown	–
**gudiv_103**	MFKTKRAKLTVGLLTVVGLITTPLIISS--CS	Sec/SPII	Yes	1	Unknown	Lipoprotein
**gudiv_159**	MVSTILIGSSIVAVAAA—CN	Sec/SPII	–	0	Unknown	–
**gudiv_162**	MKKVINKKWLGLIVGSVFVLSATAAVAAS--CN	Sec/SPII	Yes	1	Unknown	–
**gudiv_164**	MTKKKVVYSLIAGLVVGSVPASILIA—CS	Sec/SPII	Yes	1	Unknown	–
**gudiv_171**	–	–	–	0	Unknown	–
**gudiv_179**	MAGVSVIGVVAA—CA	Sec/SPII	Yes	0	Unknown	–
**gudiv_180**	MKTKKKVIISALLCSAVLVPIVGLIAS—CN	Sec/SPII	Yes	1	Unknown	Lipoprotein
**gudiv_228**	–	–	–	1	Cytoplasm	–
**gudiv_262**	MIKHKFKNKKLVVLLSLGMVAVIGATAILAS--CN	Sec/SPII	Yes	1	Plasma membrane	Lipoprotein
**gudiv_287**	MKKSLFKKELAITLGLASVAIITPIIAIA—CN	Sec/SPII	Yes	0	Unknown	–
**gudiv_331**	MVSTILVGSSIAAIAAA—CN	Sec/SPII	Yes	0	Extracellular	–
**gudiv_357**	–	–	–	7	Plasma membrane	–
**gudiv_388**	MKKFKSKKWVNYGFGLVALVGLSTSLAIA--CS	Sec/SPII	Yes	1	Unknown	–
**gudiv_398**	MKKRSKLIYFAVSTLSLSTIIGSLLIG—CT	Sec/SPII	Yes	1	Extracellular	Lipoprotein
**gudiv_402**	MKRKINKKLILFSSLITLGLSSSIIIAS—CT	Sec/SPII	Yes	1	Plasma membrane	Lipoprotein
**gudiv_410**	NKKLKSTIIFSSLFLVSIPVVIAS—CT	Sec/SPII	Yes	1	Plasma membrane	Lipoprotein
**gudiv_412**	NKRLKSTIVFSSLFLVSIPVVLAS—CT	Sec/SPII	Yes	0	Unknown	Lipoprotein
**gudiv_427**	VSKTKKKFKLLSSVLVLGLVAVVPTILA—SC	Sec/SPII	–	1	Plasma membrane	–
**gudiv_442**	MKKYQKVLLLSSFLFVVAPIVSS—CS	Sec/SPII	–	2	Unknown	Lipoprotein
**gudiv_457**	–	–	–	0	Unknown	–
**gudiv_458**	MRKQKRLLIATLISSLVVLTPIILAS—CN	Sec/SPII	Yes	1	Unknown	Lipoprotein
**gudiv_499**	MKLKKLHKQILISTSLITTFGLTSLLAA—CH	Sec/SPII	Yes	0	Unknown	Lipoprotein
**gudiv_517**	MKLKHKWLITIGSIGFISIIGFSTLASCS	Sec/SPII	–	1	Unknown	Lipoprotein
**gudiv_546**	MLKKNQIKKMLLITSTSLVSLGIVVSAVA--CS	Sec/SPII	Yes	0	Unknown	–
**gudiv_560**	MTKARKILISSFILTTIGSVSVLVAS—CS	Sec/SPII	Yes	1	Extracellular	Lipoprotein
**gudiv_633**	MKINIKFKIMASFLFLSIAPIIAVS—CS	Sec/SPII	Yes	0	Plasma membrane	–
**gudiv_635**	MKRKRIIKQAILIGAVASSISIPLLIAS—CT	Sec/SPII	Yes	0	Unknown	Lipoprotein
**gudiv_663**	MKINIKFKIMASFLFLSIAPIIAVS—CS	Sec/SPII	Yes	1	Plasma membrane	–
**gudiv_680**	MMINIKRKLMMVFLASLSTITVSSLIVA—CS	Sec/SPII	Yes	0	Unknown	Lipoprotein
**gudiv_681**	MKIKRKGIFAFASIGIVAITTTLIAS—CA	Sec/SPII	Yes	1	Unknown	Lipoprotein
**gudiv_759**	–	–	–	0	Cytoplasm	–

- Not predicted by the predictor

### The prediction analysis reveals that UdLAMPs have important characteristics both for immunobiological applications and for expression in a heterologous system

**Fig. 1:**
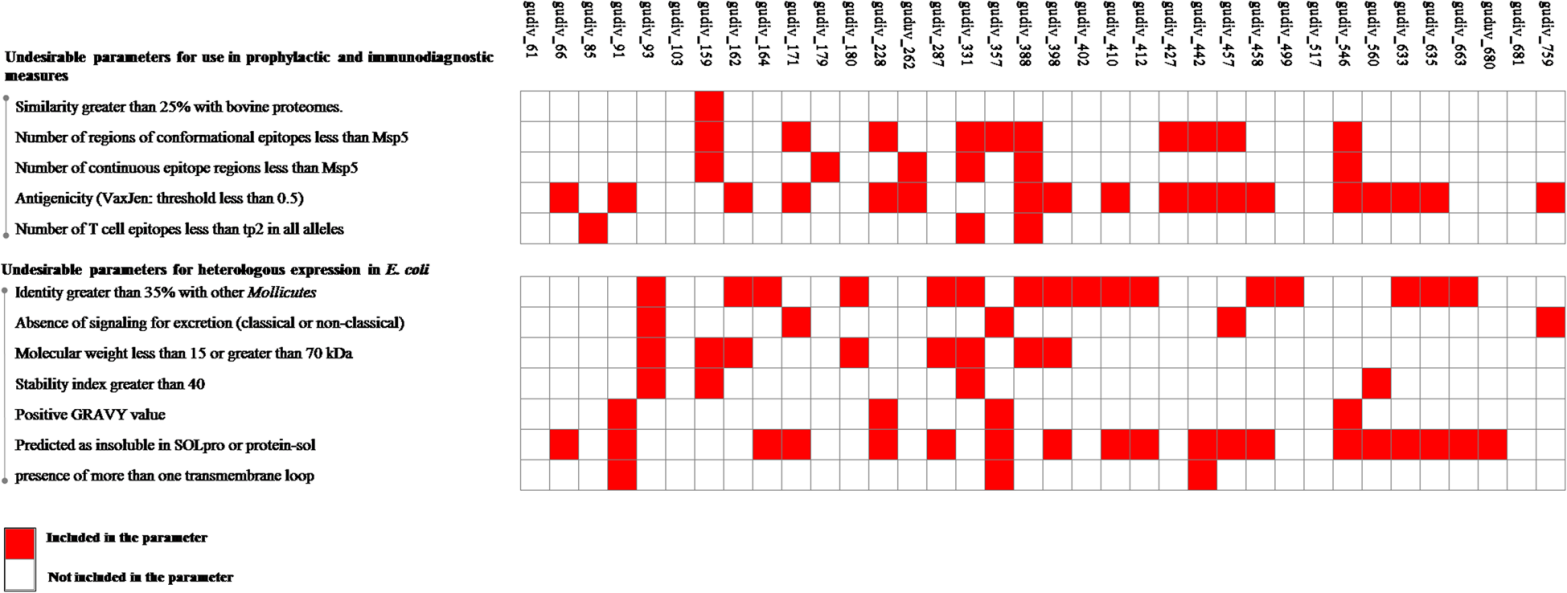
Distribution of *U. diversum* antigens according to the prediction parameters. In red, the proteins included in the evaluated parameter and in white those not included. In undesirable parameters for use in prophylactic and immunodiagnostic measures, the relevant parameters for inducing the production of specific antibodies and positive immunomodulation are evaluated. In undesirable parameters for expression in *E. coli*, predicted parameters related to the production of stable, soluble, secreted proteins and with properties that facilitate the purification process after expression were evaluated

The antigens of *U. diversum* have been classified according to undesirable properties for use in prophylactic and immunodiagnostic measures; and undesirable properties for expression in *E. coli*. The proteins gudiv_61, gudiv_103, gudiv_517, and gudiv_681 passed in all parameters, not being retained in any exclusion criteria established in this study Fig. 1. In addition, a considerable number of UdLAMPs were retained in only one or none of the exclusion criteria.

### Gene coding sequences (CDS) for LAMPs predicted as antigenic are present in strains from different Brazilian states

**Fig. 2:**
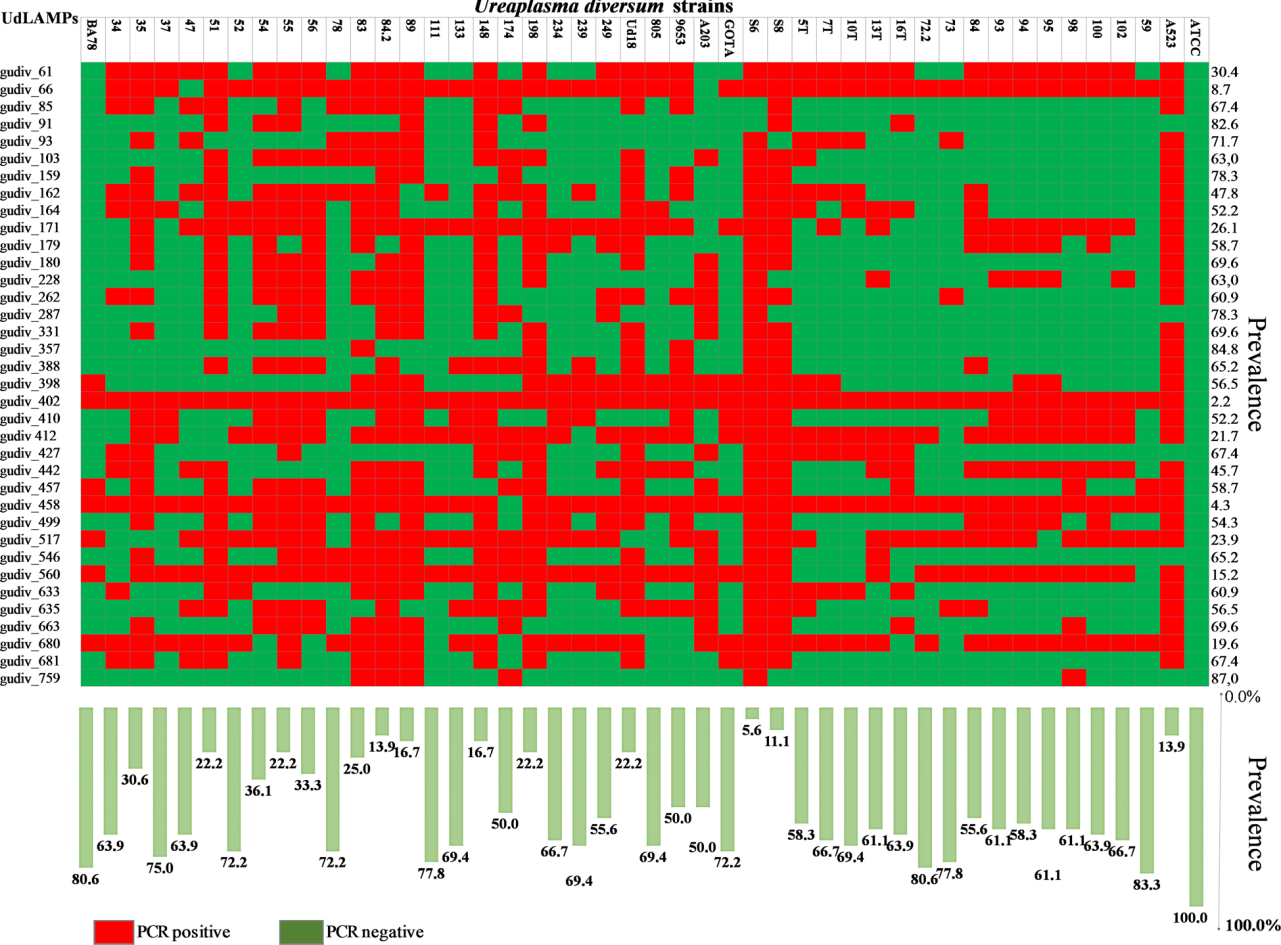
Distribution of *U. diversum* antigens in 46 strains, one standard strain (ATCC) and 45 isolates from different Brazilian regions. The diagram shows detection by PCR of the antigens in each strain (in green PCR positive and in red negative). On the bottom is the total percentage of antigens that each strain carries in its genome (based on PCR results) and on the left is the percentage of strains carrying the coding sequence for each antigen individually

**Fig. 3:**
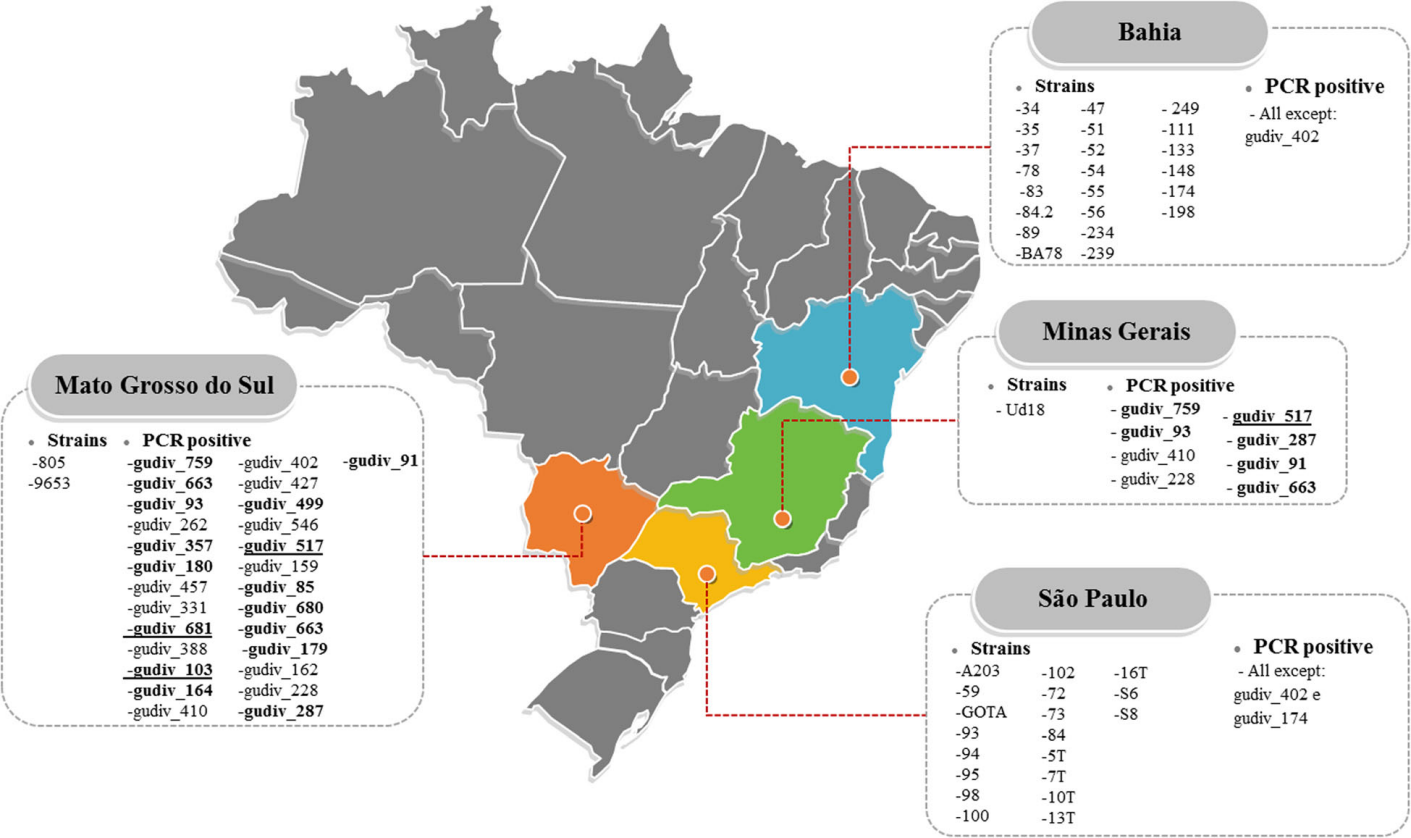
Distribution of *U. diversum* antigens in different Brazilian states. The Brazilian states studied are represented in highlighted colors. In each state, the isolated strains and the set of UdLAMPs positive by PCR are used. In bold and underlined are UdLAMPs that (by prediction) showed good characteristics for immunological studies and expression in *E. coli*, not being retained in any of the exclusion criteria in Diagram 1. Only in bold, the antigens that, although they did not pass all criteria, were maintained in only one or two of the undesirable parameters for use in prophylactic and immunodiagnostic measures in Fig. 1. The figure was acquired at Wikimedia Commons (https://commons.wikimedia.org/wiki/File:Brazil_Map-1.png) and was adapted using Adobe Photoshop CS6 version 13.0.6 × 64

To verify the distribution of *U. diversum* antigens in different Brazilian states, the presence of genes for LAMPs in 46 *U. diversum* strains was investigated by PCR. Table 7 lists the primers constructed. All antigens were detected in strain ATCC 49782. The lowest and highest percentage of amplified antigens (not considering the ATCC strain) occurred for strains S8 and 59, respectively, 5.6 and 83.3% (Fig. 2). Regarding antigens, the highest prevalence was gudiv_759, gudiv_357, and gudiv_91 detected in 87, 84.8, and 82.6% of the strains, respectively. In contrast, the least present were gudiv_402 (2.2%) and gudiv_458 (4.3%). The presence of antigens varied in the strains isolated from the states studied (Fig. 3). In Bahia, the state with the highest number of strains, a total of 35 antigens were detected by PCR. The only strain in Minas Gerais tested positive for seven proteins. Isolated representatives of Mato Grosso do Sul (805 and 9653) had 27 antigens. In São Paulo, all 13 strains were PCR positive for 34 proteins.

**Table 7:** Primers for amplifying UdLAMPs using conventional PCR

UdLAMPs	Forward	Reverse	Fragment length (bp)
**gudiv_061**	CAGTAAGTGTTGTTGCTG	GTTACCGAAGTCTTGTCC	797
**gudiv_066**	AGCGTTGCCTTAGTTAGT	TTAATCCGTCCCACATTG	965
**gudiv_085**	CAGGAAGTGCTACAGTTG	ACTCATCATTTACCACCT TC	421
**gudiv_091**	CTGAAACCGCTTTAACAAG	ACAACAAGCCGACTAAATC	652
**gudiv_093**^a^	GGCTCAAGTAGTGAAGAGAAG	GCAAATGGAATTGGATGTAC	680
	TTCTGAACCTGAACCACAA	CTAATTCACGACTGCCTT	937
**gudiv_103**	GTACCTAATCTCAATCAAGC	CAACTAAGTCAACACGAG C	307
**gudiv_159**	CACCTAATCCATCAAAGAAC	GTTTGTAGTAGAGTTGCCTA	260
**gudiv_162**	CTCAGTAACTACACCACTT	TGCTTTACCTGTACGGAAT	298
**gudiv_164**	GTAGTAGGTTCAGTTCCT G	GATCAGAAGATAGCGATCAG	732
**gudiv_171**	CCAGATGGTAATGCTGAAC	CTACTCATGCTCTTAGTTC	547
**gudiv_179**	GCGAAGATCCTAAAGCAAT	CGAACCTGAAGTAATAAGG	379
**gudiv_180**	GCTTGGAAGACAACTCTAA	TTCTAGCACCTCAGGTAG	930
**gudiv_228**	GAGGAACTTTTAGTGATCCA	CATGGTTATACAAAGGGGTG	125
**gudiv_262**^a^	CATTAGGTATGGTTGCTGTA	GTTTGATCGGCTCTTCTG	252
	CATTAGGTATGGTTGCTG	CTTCTGGCATCGGATTTG	543
**gudiv_287**	TGAGCAACCAAATACTGAAG	GCTTGAGTTGATGTGTTTG	312
**gudiv_331**	GGTAAGCACGATCCTTGT	TTTGTTTGCGGCTGAATC	178
**gudiv_357**	GGTAATTGGTGGTGTAATAG	AAGTGATTCAGGAGTTGC	232
**gudiv_388**	CTATGGGTTTGGATTAGT	CTTTTCTTTAGCAGCTTC	187
**gudiv_398**	CGTTATCAATACCACGACTTC	TGGAATATGTCCTGATCG	351
**gudiv_402**	GGTAGAGGTAGTGGTTCA	AGCAGATGGATCTTCGTAAT	945
**gudiv_410**	CACTAGAACCAGCAAAACC	CCAAAATATCAGTCCGATCAG	819
**gudiv_412**	ACCACTTGTAGCACTAGA	CTAAAATATCAGCCCGATCAG	830
**gudiv_427**	TTAGGATTGGTTGCTGTTG	GATTGTTGTGGGTGAAAATC	509
**gudiv_442**	CTACCAGATAGTATTGCTC	GGTGGACTTGTTAATGTATC	809
**gudiv_457**	CAGAAGAATCACTAGAGC	CTGCTGGGTTATCACTTC	360
**gudiv_458**	CCAACCAACTCCTAAACTAG	GCACTCCAAGTGATTCATC	482
**gudiv_499**	TAATCTTCCAACCCATCAAG	CTTCTTTTGCTGTATGAGC	572
**gudiv_517**	AACCAACTTTGAGCAAGC	GCTGCTTTAGAAAAGATAG	321
**gudiv_546**	CAGTTGCTTGTTCACAAC	GGCTTGGTTTGGTTCAAA	266
**gudiv_560**	GGATCAGTTAGTGTACTTGTTG	TTAGCAAAGGTTGGATCTTC	344
**gudiv_635**	GCAGTTGCTTCATCTATCT	CTAGTCTTGCTACCTTATC	607
**gudiv_663**	CGCTACAACTATGACTGAT	AATGGCTGACCAAATTGTG	243
**gudiv_663**	CTGATCCTACAGTGGTTAAAC	CCATCAAAGATGAAGTCTTG	607
**gudiv_680**	GACAGAGTCTCCAAAACC	TCTAACTGTTTCTTCATTAGGG	547
**gudiv_681**	GATTAGTTCAAGTGGTGAAG	ATCATCAACAGCAGTCTT G	346
**gudiv_759**	GCTGATGAAGGATATTATGG	GCAAGTGATAGATCGTTTG	749

^a^Two pairs of primers were designed for gudiv_93 and gudiv_262

## Discussion

*Mollicutes* lipoproteins are important virulence factors associated with pathogenesis in the reproductive and respiratory tract of infected hosts [10]. In this study, the lipoprotein gudiv_159 had 29% similarity with the Tektin-1 protein from the *Bos taurus taurus* proteome. For the other UdLAMPs and proteins from other bovine subspecies, all similarity values were less than 12%. Similarity values greater than 25% are relevant when assessing immunological aspects [11]. The similarity between virulence factors and host proteins can make it difficult to develop an adequate immune response, or even generate cross-reaction events with autoantibody production during infection [12]*. Mycoplasma hominis*, *M. fermentans* and *M. arthitides* are species of *Mollicutes* often found in patients with autoimmune diseases [10].

A protective immune response with the production of effector cells and antibodies able to recognize epitopes of an infectious agent are essential for fighting infection. Conformational epitopes represent the majority of B cell epitopes (about 90%). However, conformational epitopes usually contain one or a few stretches of linear epitopes [13]. In the prediction, we found that all 36 UdLAMPs have conformational and linear epitopes for B lymphocytes and are predicted as antigenic (VaxiJen predictor). A considerable number of regions of conformational and linear epitopes were greater than or equal to the values for Msp5, one of the main surface proteins of *A. marginale*, known for its ability to induce antibody production during cattle infection [14]. The presence of these epitopes points to these molecules as agents capable of stimulating the development of a humoral immunological response.

*U. diversum* can also behave as an optional intracellular pathogen [15]. Thus, the possibility of UdLAMPs being processed and presented via MHCI can lead to cellular response activation. In this study, epitopes binding to bovine MHCI alleles were predicted in several UdLAMPs. Furthermore, 33 LAMPs had connections equal to or greater than the *T. parva* Tp2 antigen in all studied alleles. Tp2 is recognized for stimulating CD8^+^ T cells during bovine *T. parva* infection [16]. The studied alleles represent cattle destined for the different livestock sectors. Five alleles representing *Bos taurus taurus* (BoLA-6 * 01301, BoLA-2 * 01201, BoLA-3 * 00201, BoLA-1 * 02301 and BoLA-6 * 04101), two alleles representing *Bos taurus indicus* (Bola - T5, BoLA-3 * 00101) and an allele (BoLA-T2C) belongs to a hybrid [17]. Taurine breeds are predominantly found on dairy farms and Zebu cattle are mostly used for meat production [18]. Bovine hybrids are usually produced to align the commercial and management characteristics of both subspecies [19]. In this case, our prediction data reveal that a considerable number of UdLAMPs can interact with MHCI alleles of cattle destined for different activities in the livestock sector, reflecting in activation of inactivation immune response.

The identity analysis of UdLAMPs with proteomes of other microorganisms capable of infecting cattle is a useful initial approach for studies aimed at using these antigens or antibodies produced in immunodetection tests. We found that the proteins gudiv_103, gudiv_159, gudiv_171, gudiv_228, gudiv_517, gudiv_546, gudiv_680, and gudiv_681 did not present a significant identity with the proteins of other important *Mollicutes* that infect bovine. In contrast, 25 proteins showed an identity greater than 30%. According to Rost [20] above a cutoff point of 30% identity, 90% of the pairs are homologous. The low identity between proteins of different infectious agents from the same host is related to good specificity when considering detection tests [21]. Thus, *U. diversum* proteins with low identity may represent specific targets for use in immunodiagnostic techniques in detecting this pathogen.

In addition to the prediction of immunobiological properties, the prediction of properties favorable to expression in a heterologous system can contribute to the broad scale of a protein biological target. Some physicochemical properties influence the state of solubility, the formation of inclusion bodies or proteolysis of the heterologous peptide [22]. In this study, the protein PM ranged from 9.0 to 240.2 kDa. Proteins with PM between 70 and 60 kDa are well tolerated when *E. coli* is used as an expression system; however, proteins with very high PM are not adequately expressed in these bacteria, and are, therefore, degraded or structured in the form of inclusion bodies [23]. Small peptides (about 10 kDa) are also difficult to express in stable form due to improper folding, so they are often subject to proteolytic degradation [24].

Our analyses also showed that only gudiv_93, gudiv_159, gudiv_331 and gudiv_560 had an instability index greater than 40 and, therefore, all the others (with an index below 40) were considered to be stable [25]. Most of the proteins were GRAVY negative, which is related to hydrophilicity [26]. Greater hydrophilicity implies a greater capacity to form hydrogen bonds with water molecules and, consequently, greater solubility [27]. Sixteen proteins were predicted to be soluble in the two predictors used in this work (Solpro and proteinSol) and only two proteins had more than two predicted transmembrane loops. Transmembrane loops are hydrophobic regions that reduce solubility [28]. Expression in the soluble form is desirable, because to obtain soluble proteins from insoluble forms, a series of processing steps that involves the use of strong denaturants followed by renaturation is inevitable [29]. Even so, these additional steps do not guarantee the production of soluble and functional proteins.

The presence of specific markers capable of directing heterologous peptides to the extracellular medium in an expression system also contributes to the subsequent steps in the production of recombinant proteins [23, 30, 31]. Here, we show that more than half of the studied proteins were predicted to possess a SP recognized by sec/SPII and consequently likely a lipoprotein capable of being expressed and exported to the extracellular medium by *E. coli*. The presence of a SP for a classical secretory pathway or markers for secretion by a non-classical pathway facilitates the transport and secretion of the transcript into the extracellular compartment. Secretion in the extracellular medium simplifies purification processes, protects heterologous proteins from proteolysis, decreases endotoxin levels, and improves biological activity and solubility [32].

Bacterial proteins with good properties both for stimulating the immune response and for cloning and expression in a heterologous system are desirable targets for biotechnology [30]. In this study, the use of a filter with exclusion criteria based on the prediction data (In Diagram 1) showed that gudiv_61, gudiv_103, gudiv_517, and gudiv_681 are the ULAMPs most promising for immunobiological applications and for expression in *E. coli* as a heterologous system. However, the fact that an antigen does not meet all the requirements of Diagram 1 does not rule it out as a target for immunobiological studies or expression in a heterologous system. Depending on the type of analysis, proteins having good immunostimulatory properties, but with properties that hinder expression in *E. coli* could be expressed in other expression systems [33], or even in *E. coli* through fusion with proteins (tag) that increase the size of the transcript or improve solubility, reduce growth temperature, use of weak promoters and use of low concentrations of inducer [24]. Very large proteins or with many transmembrane loops could be studied by producing multiepitope chimeric proteins [34]. Finally, there is also the possibility of using expression systems entirely in vitro [35]. However, these alternatives increase the costs of the process; therefore, the inclusion of prediction in the planning stages of works that intend to express proteins can reduce project costs in addition to providing a theoretical forecast of bench tests.

In this work, the PCR detection of 36 UdLAMPs in isolates from *U. diversum*, from different regions of Brazil, warns of potential damage to livestock that *U. diversum* can cause, because in addition to immunomodulation, studies suggest that LAMPs are involved in adherence and invasion and cell apoptosis [2, 7, 15, 36]. Strains representing the four evaluated states (Bahia, Minas Gerais, São Paulo, and Mato Grosso do Sul) presented proteins with interesting properties for immunological stimulation (Diagrams 1 and 3). These data corroborate with other studies that show that *U. diversum* induces variable immune responses in vivo and in vitro [7, 37].

## Conclusion

It was demonstrated that the *U. diversum* genome has CDS for molecules with potential for application in immunodiagnostic or immunoprophylactic tests and expression in *E. coli* as a heterologous system. PCR screening of antigens on strains from different states revealed that UdLAMPs have a heterogeneous distribution in different regions of Bahia, Minas Gerais, São Paulo, and Mato Grosso do Sul. In this study, 34 of the 36 UdLAMPs studied were noted in the genome as UdLAMPs and that many of them have signaling of typical lipoprotein secretion. It is well described in the literature that *Mollicutes* have ingenious molecular mechanisms to change parts of these molecules; however, this initial study contributes to understanding the virulence factors of *U. diversum* and provides a series of data and approaches that can be used in studying these pathogens.

## Methods

### Access to genes and analysis of similarity with bovine proteomes

The CDS and peptide sequences of 36 UdLAMPs, strain ATCC 49782, were accessed through the Manatee database (https://manatee.igs.umaryland.edu. The DNA sequences also are available in the GenBank: CP009770). Similarity analyses between proteins of *U. diversum* and proteomes of bovine subspecies (*Bos taurus taurus*, *Bos taurus indicus* and the hybrid *Bos taurus* x *Bos indicus*) were performed using the BLASTp tool accessed on the server https://www.ncbi.nlm.nih.gov. The proteomes were accessed on the BLASTp platform itself through the UniProtKB/Swiss-prot (swissprot) database under taxonomy IDs 9913 (*Bos taurus taurus*, protein count: 37513), 9915 (*Bos taurus indicus*, protein count 1243) and 30,522 (hybrid *Bos taurus* x *Bos indicus*, protein count: 42151).

### Mapping of B lymphocyte epitopes and antigenicity prediction

The CBTOPE v1.0 server (available at http://crdd.osdd.net/raghava/cbtope/) was used to predict discontinuous (conformational) epitopes of B lymphocytes. A threshold of − 0.3 was used, and on the probability scale (0–9) amino acids with values greater than four were considered conformational epitopes. This server has a data set with non-redundant protein chains consisting of antibody interacting residues of B cell epitopes [38]. To predict continuous epitopes, the primary protein sequences were analyzed in the BepiPred v2.0 software (http://www.cbs.dtu.dk/services/BepiPred/), a predictor trained only with data, present in your internal database, from epitopes derived from crystallographic structures. Amino acids with thresholds greater than 0.5 were considered linear B cell epitopes [13]. The protein sequences were also submitted to the VaxiJen v2.0 server (http://www.ddg-pharmfac.net/vaxijen/VaxiJen/VaxiJen.html); this predictor allows classifying antigens without using the sequence alignment feature. All proteins predicted to score above thresholds (0.5) were classified as antigenic. The prediction of B cell epitopes and antigenicity was also performed for the Msp5 ESXA_MYCBO peptide from *A. marginale* accessed at NCBI under ID number AY527217.1.

### Mapping of TCD8^+^ lymphocyte epitopes and identity analysis with proteomes of other *Mollicutes*

The prediction of binding to MHCI with peptide windows with 9 amino acids, was performed using the server NetBoLApan v1.0, accessed at http://www.cbs.dtu.dk/services/NetBoLApan/. A standard threshold of 0.5% was used for strong bonds and 2% for weak bonds; finally, the number of strong and weak connections were added and expressed in absolute numbers. The NetBoLApan v1.0 was trained on a peptide dataset with binding affinity to BoLA molecules [39]. The alleles used in this study were BoLA-6*01301 (HD6), BoLA-2*01201 (T2A), BoLA-3*00201 (JSP), BoLA-1*02301 (D18.4), BoLA-3*00101 (AW10), BoLA-6*04101 (T2B), BoLA-T2C and Bola–T5. In this set of alleles there are representatives of three bovine subspecies (*Bos taurus taurus*, *Bos taurus indicus* and the hybrid *Bos taurus taurus x Bos taurus indicus*), thus including cattle involved in various livestock activities [17]. The same analyses were performed for *Theileria parva* Tp2 antigen.

The BLASTp was used for identity analysis of 36 UdLAMPs with proteomes of *M. bovis* (831 protein count and taxonomy IDs: 28903), *M. canadense* (481 protein count and taxonomy IDs: 29554), *M. bovigenitalium* (677protein count and IDs taxonomy: 1188235), *M. bovirhinis* (720 protein count and taxonomy IDs: 29553), and *M. dispar* (712 protein count and taxonomy IDs: 86660).

### Prediction of secretion and subcellular localization

Prediction of classical secretion and identification of SP were performed on the SignalP v5.0 server available at http://www.cbs.dtu.dk/services/SignalP/ and DOLOP, a server that uses SP characteristics to predict lipoproteins -https://www.mrc-lmb.cam.ac.uk/genomes/dolop/. The SecretomeP v2.0 web server (http://www.cbs.dtu.dk/services/SecretomeP) was used to predict non-classical secretion. Predicted values equal to or greater than 0.5 (threshold) were considered indicative of secretion. Protein sequences were also subjected to the prediction of subcellular location in the PSORTb v3.0.2 software (http://www.psort.org/psortb/) using suggested settings for *Mycoplasma* spp. TMHMM v2.0 (http://www.cbs.dtu.dk/services/TMHMM/) was used to verify the presence of transmembrane loops.

### Investigation of physical-chemical parameters

The physicochemical properties of UdLAMPs including aliphatic index, PM, GRAVY, and instability index were obtained in ProtParam using the ExPASy server at http://web.expasy.org/protparam/. The solubility of heterologous peptides after *E. coli* overexpression was predicted by the server SOLpro (http://scratch.proteomics.ics.uci.edu/) and Protein-Sol, accessed at https://protein-sol.manchester.ac.uk/.

### Filter properties related to immunomodulation and expression in a heterologous system

*U. diversum* antigens were classified according to two parameters: 1) Undesirable parameters for use in prophylactic and immunodiagnostic measures; in which prediction results for similarity with bovine proteomes were evaluated, number of conformational and continuous epitope regions for B lymphocytes, antigenicity, number of T lymphocyte epitopes (BoLA allele ligands) and identity with other *Mollicutes* that infect cattle; 2) undesirable parameters for expression in *E. coli*; in which predicted parameters related to the absence of signaling for excretion by classical or non-classical pathways, protein size, stability index, GRAVY, solubility and presence of transmembrane loops were evaluated.

### Obtaining, cultivating and extracting DNA from *U. diversum*

*U. diversum* ATCC 49782 and 45 isolates were provided by the Mycoplasma laboratory of the Institute of Biomedical Sciences - University of São Paulo (USP). Some strains were isolated from cows that had granulomatous vulvovaginitis, and others were isolated from the semen of healthy bulls. The isolates were obtained from four states: 19 isolated in São Paulo (farms 1, 2, 4, 8 and 9), 2 isolated in Mato Grosso do Sul (farm 3), 1 in Minas Gerais (farm 6), and 22 in Bahia (farms 10, 11, 12, 13). One milliliter of each sample previously-stored in UB medium was grown in 9 ml of the same medium at 37 °C for 24 to 48 h [7]. After growth, bacterial DNA was extracted using the NucleoSpin kit (Macherey-Nagel, Germany) following the manufacturer’s instructions. After growth, bacterial DNA was extracted using the NucleoSpin kit (Macherey-Nagel, Germany) following the manufacturer’s instructions.

### Primer construction, PCR, and electrophoresis

The genomic sequences coding for the 36 antigens of *U. diversum* were used to design the primers by the servers https://www.idtdna.com/calc/analyzer and https://www.bioinformatics.org/sms/revcomp.html. Important criteria for the efficiency of primers such as size (18 to 22 bp), melting temperature (52 to 58 °C), and G + C content (40 to 60%) were taken into account. After selecting the best pairs of primers, similarity analysis was performed by the BLASTn server (https://blast.ncbi.nlm.nih.gov/Blast.cgi) to confirm the specificity of the primer sequence for *U. diversum*. The 46 strains were evaluated for the presence of genes for UdLAMPs using PCR. The amplifications were performed with a total volume of 25 μl containing: 1 μl of DNA, 10x PCR buffer (10 mM Tris – HCl, pH 9.0; 50 mM KCl), 1.5 mM MgCl2; 200 μM dNTP, 50 pmol of each primer and 1.5 U of Taq DNA polymerase (Invitrogen®, Brazil). All genes followed the initial denaturation of 94 °C for 5 min, followed by 35 thermal cycles of 94 °C for 30 s, 54 °C for 30 s, and 72 °C for one minute, concluding with a final extension 72 °C for 5 min. The reaction products were analyzed by electrophoresis on 1.5% agarose gel, stained with 2.5 μl ethidium bromide (10 mg / ml), visualized and photographed under UV light. A molecular weight marker (Invitrogen®, Brazil) - was used as a standard to assess the size of the amplified fragments.

## Supplementary information

**Additional file 1: Table S1.** Sequences of the most significant B lymphocyte epitopes (highest thresholds in the Pepipred 2.0 predictor) accompanied by the position of the respective epitope in each UdLAMP.

**Additional file 2: Table S2.** Sequence of the most representative CD8+ T lymphocyte epitopes and respective position in each UdLAMP predicted using NetBoLApan v1.0. Peptides with an 8-amino acid window of the BoLA-1 *02301, BoLA-3 *00201, BoLA-2 *01201 and BoLA-6 *01301 alleles are represented.

**Additional file 3: Table S3.** Sequence of the most representative CD8+ T lymphocyte epitopes and respective position in each UdLAMP predicted using NetBoLApan v1.0. Peptides with an 8-amino acid window of the BoLA-3 *00101, BoLA-4 *04101, BoLA-T2C, and BoLA-T5 alleles are represented.

## Data Availability

The DNA sequences generated and/or analyzed during the current study are available in the GenBank repository, Accession: CP009770. The others datasets used and/or analyzed during the current study are available from the corresponding author on reasonable request.

## Abbreviations

Aa: Amino acids
ATCC: American Type Culture Collection
BoLA: Bovine lymphocyte antigen
CDS: Gene coding sequences
dNTP: Deoxynucleotides
G +C: Guanine-cytosine content
GRAVY: Grand average of hydropathy
IgG: Immunoglobulin G
KDa: Kilodalton
LAMP: Lipid-associated membrane protein
MBA: Multiple band antigen
MHCI: Major histocompatibility complex class I
MIB: *Mycoplasma* Ig binding protein
MIP: *Mycoplasma* Ig protease
Msp5: Surface protein 5 from *A. marginale*
PCR: Polymerase Chain Reaction
PM: Molecular weight
SP: Signal peptide
Tp2: *T. parva* 2 antigen
UB: Ureaplasma base
UdLAMPs: Lipid-associated membrane protein of *U. diversum*
