# Supplementary material for: Immunoinformatics and analysis of antigen distribution of Ureaplasma diversum strains isolated from different Brazilian states
Source: BMC Vet Res. 2020 Oct 7;16:379. doi: 10.1186/s12917-020-02602-1 (PMC7542862; doi:10.1186/s12917-020-02602-1)
Supplement: Supplementary file 1 — Additional file 1: Table S1. Sequences of the most significant B lymphocyte epitopes (highest thresholds in the Pepipred 2.0 predictor) accompanied by the position of the respective epitope in each UdLAMP. [file 12917_2020_2602_MOESM1_ESM.docx]

**Additional table 1.** Sequences of the most significant B lymphocyte epitopes (highest thresholds in the Pepipred 2.0 predictor) accompanied by the position of the respective epitope in each UdLAMP.

| **LAMPs** | **Position** | **Epitopes** | **LAMPs** | **Position** | **Epitopes** | **LAMPs** | **Position** | **Epitopes** |
| --- | --- | --- | --- | --- | --- | --- | --- | --- |
| gudiv_61 | 27-41 | ACNNENKKGTTPSTG | gudiv_180 | 548-556 | SAITNNSSN | gudiv_442 | 31-45 | SIALKSTVSFITDEI |
|  | 42-56 | VQTQTQVKKGTTPST |  | 566-574 | DIELGLENI |  | 46-60 | NSLTTKYIDQRTRFT |
|  | 58- 65 | GVQTQTQVA |  | 582-587 | DKLEKT |  | 61-75 | HLHNKNSQINLKQNQ |
|  | 99-114 | LLEAQKDVFDKDKVRY |  | 600-606 | NPNGPNK |  | 76-80 | KEAVF |
|  | 121-133 | KASTLKVQNTNGK |  | 653-667 | DSSMQYRTTIGKWID |  | 87-101 | DKTYEKQLRKQIAEA |
|  | 156-170 | VLINKLSYNPKTWNE |  | 668-681 | INSDKVELNDIGT |  | 102-109 | VLEKNHLA |
|  | 171-181 | FVKAEISTELD |  | 687-698 | KKPDTNHMLSYV |  | 116-130 | GNDHFFSYDKEIISI |
|  | 204-211 | EQETEKQA |  | 705-717 | KGRDIDKDVKYYN |  | 147-161 | KNDKNNISKQVNVDQ |
|  | 219-227 | VKLDEALP |  | 725-739 | SSMQYRLVNSSDWKE |  | 162-172 | FLTKNYSSYFL |
|  | 232-246 | WMQGVDLGATDKKPW |  | 740-750 | ITTNRLSNLKP |  | 177-189 | VAKLIKIDDLNGD |
|  | 247-263 | NHIKKDEKGKRVKSNKE |  | 759-770 | PNGTQLASDPVK |  | 197-211 | KDQSHTDIGLTKEGM |
|  | 264-279 | DRMNFVNDNKLDLTKE | gudiv_228 | 23-37 | VKQIRGTFSDPFSSK |  | 212-226 | QQMIKYKKLSNFITT |
|  | 280-287 | LTKEGQDFGNAV |  | 38-46 | TPDADTLDK |  | 233-247 | ELEKKNSSRSVNYDL |
|  | 301-316 | DFNDNATKRKGLEERN |  | 47-55 | SIWQKQFSD |  | 248-262 | YEHLLTNKEWKALHD |
|  | 317-334 | KVLDDLKLFGNSDANVK |  | 81-94 | KIRPRTNKMSVEEY |  | 273-279 | KNEEIKI |
|  | 335-351 | KGVRYEIKEDGSVIKKEQ |  | 101-110 | KINSFKFILK |  | 291-305 | DTLTSPPPIDTENDW |
|  | 371-388 | DALHKNIYKMKDSEKLEL |  | 116-123 | IPGVKEFN | gudiv_457 | 6--17 | WDGLFRDSIPLA |
|  | 393-401 | LKSKLWWEP |  | 191-205 | KSIHHNGILNETVEI |  | 51-65 | HELFQETSEESLERI |
| gudiv_66 | 35-44 | TNDLNNKPET |  | 206-219 | NVDNKHLDKTLDVD |  | 66-80 | RSESIDNGIFFKPKS |
|  | 53-67 | DFIYSFNNQSFINQL | gudiv_262 | 34-48 | DNKNTPVKKVENKNK |  | 92-100 | DNGIVDEKN |
|  | 68-76 | SDDDQKQIH |  | 49-63 | NVTVEANKKTNSDVS |  | 121-135 | KFYTDNTKKTLTKKR |
|  | 123-129 | DKKTSSY |  | 64-78 | KNQTNQPKQDNNQNL |  | 148-162 | LQQHPEQLLNNFNIR |
|  | 140-154 | NDLGNNLDAITKPQF |  | 79-93 | EKNNQQTTPKVEKDS |  | 163-167 | KYTEV |
|  | 155-169 | YTPTKDNSLPTHINN |  | 94-108 | SEEPIKPQINLKPDD |  | 169-183 | QFIRSDNPAVSSTAQ |
|  | 170-184 | PQPYWFLNKEEIIKK |  | 109-123 | KKDQQDQQDQQPETG |  | 184-194 | LIFNDEYGGNF |
|  | 205-219 | AKTATNNQVLSAFSF |  | 124-138 | LNQKEQGNPKDPETK | gudiv_458 | 29-43 | NQANRSKDQATKQKN |
|  | 232-242 | NQKSEVNDGFA |  | 139-153 | KEEPAPEVKTPEPNT |  | 44-58 | TNSIKRLDDLFDQSS |
|  | 249-264 | LIQQKEIDHEVELATF |  | 154-168 | SEQNGKPEVKQEGMQ |  | 59-73 | TNPKANSNDQENEPL |
|  | 278-288 | SNQLPDVKKIT |  | 169-183 | EKNEKDSMGLGGQSN |  | 80-94 | IKFNKIDLDQNQPTP |
|  | 297-305 | LDEFRYDVK |  | 184-198 | TQQNGENSNPMPEEK |  | 95-109 | KLESTKPNPPKNQLE |
|  | 313-327 | FATDQSLLSIKHHQS |  | 199-213 | MQGDQADQEQKVPEE |  | 119-128 | IQNQDNDPNF |
|  | 328-333 | DKTNTK |  | 214-228 | RKLTRDELLIGTKKI |  | 139-148 | ATNQEELKKE |
|  | 336-339 | QQWTENNVGRINHIN |  | 229-243 | LDWMLENEKKKLITE |  | 184-190 | TVNNYKQ |
|  | 351-357 | PKLSSII |  | 244-258 | EQKQLGFYRKPGSVK |  | 197-201 | NKSQL |
|  | 370-384 | FDQTFEPENQSGIKH |  | 259-273 | DEDVYDVDFGKLFNT |  | 213-227 | VVKPNNNQNKLINQS |
|  | 385-389 | CHADG |  | 274-278 | TEPDK |  | 228-233 | DQLYPN |
| gudiv_85 | 34-48 | KTKGGENKKTTPASP |  | 286-299 | SDYKNSRNLLNVKK |  | 240-246 | ENKNNDE |
|  | 49-63 | TNKQTAAPSVSLDSN | gudiv_287 | 142-154 | AYDTNTSTQATSL |  | 262-268 | NESLQKQ |
|  | 64-73 | SKLTKRADNK |  | 159-173 | EYTNKFFETKPIYAT |  | 301-315 | DHSKLETYKKILTQN |
|  | 92-103 | LTSQSTNTNNSL |  | 174-188 | NFVNQEEFSAFENKT |  | 316-320 | FNYEP |
|  | 122-130 | LDENTSYSL |  | 189-196 | LQQKGHEA |  | 332-346 | QVVDYKKYQKNQKDE |
|  | 139-153 | NTDTNPTNVELSQSL |  | 198-212 | QNLDKFNSEQWYEFE |  | 357-365 | TYTNQINQP |
|  | 161-176 | LKSNEEHDHSSHMGHN |  | 224-238 | YSVNGTRNFNPLQDY |  | 376-387 | LTKSQTKQNLTS |
|  | 177-192 | GSGSSPIVGNGNTNQK |  | 239-253 | DNGLKVTDQQKQFFD |  | 396-409 | WNTFEQQEFAINNS |
|  | 193-208 | GKEIEGGAEGTNKGDR |  | 254-268 | NKAKELSQETYDKAA |  | 417-426 | TNLMQTVDFS |
|  | 209-224 | DQLQGDQAPGNPQPDA |  | 269-283 | LKGFTLPIYKENGNE |  | 437-451 | NDKLPENDNNSNHLS |
|  | 225-243 | GASKTGEYKIGFKKVI |  | 284-298 | VLGLSTKEGNPSLQH |  | 452-463 | GWNLEAAKINLA |
|  | 244-249 | YQEGGK |  | 299-313 | SSLASNVTRATGTPR |  | 472-478 | NNNESYS |
|  | 270-283 | NSAGTQINTKNATI |  | 214-223 | ILPGQKYKDI |  | 485-492 | NKRSKKYK |
|  | 299-313 | SSIKPNKQYRLSRID |  | 335-341 | PIQGQTR | Gudiv_499 | 34-54 | DQLSKYEQIPTLKEKVQLNRI |
|  | 314-328 | ITDKQDKKTEIRNFD |  | 377-391 | LTDLVGISFTFLKPE |  | 64-70 | NQYVNLP |
|  | 329-343 | PTISGEITSVPQRGA |  | 392-406 | AKLQKTQKLIKLDED |  | 101-108 | SEHTRTYT |
|  | 344-358 | GKQQADSPSVQSNPG |  | 407-419 | YTILNIPTTNEQK |  | 112-126 | GGFKDAKDDRIQKAN |
|  | 359-373 | STSTNTGDSSKGSGV |  | 431-445 | LNSKPSDFLTPAQAD |  | 127-141 | QTYLNDPKNQTTLFK |
|  | 374-382 | WFENNKPEV |  | 446-451 | KYKDTD |  | 142-156 | DIELVNTKVEDLTVD |
|  | 412-427 | VKGSSSINNTAKTEDL |  | 465-479 | EIMKIKESFIQQGGQ |  | 157-163 | DALKLSS |
|  | 428-440 | ADIEKVEQGKGTK |  | 480-494 | TEDKKEKIESPAELA |  | 168-174 | VQNDNKE |
|  | 454-462 | TAETDGSKK |  | 495-509 | KKVTNEYATNKENHI |  | 183-189 | TKDTSNP |

**Additional table 1.** Sequences of the most significant B lymphocyte epitopes (highest thresholds in the Pepipred 2.0 predictor) accompanied by the position of the respective epitope in each UdLAMP.

**Continuation**

| **UdLAMPs** | **Position** | **Epitopes** | **LAMPs** | **Position** | **Epitopes** | **LAMPs** | **Position** | **Epitopes** |
| --- | --- | --- | --- | --- | --- | --- | --- | --- |
| gudiv_85 | 469-483 | EWLGEDLTPVKSEDL | gudiv_287 | 510-524 | KFLKTSYLQDFNKID | Gudiv_499 | 201-206 | GVNDQL |
| gudiv_91 | 5--19 | SHSLWLESFIYNTQT |  | 525-534 | GPILDNTKWK |  | 212-226 | TKKINNLLTVDQKKA |
|  | 19-34 | NQSNTETVANDSHWS |  | 546-560 | GAQEIDKTTERAGNA |  | 227-241 | KTNNLSIATNTQYHV |
|  | 68-81 | VKWKSESGNIKSKK |  | 561-575 | LWTNSFYILLQDHQR |  | 242-256 | APTISDELKKAHTAK |
|  | 124-134 | RYVGAGSHAEA |  | 576-590 | NDQTYQNLGNLLSKH |  | 257-272 | EATEHKEWFKFDATNY |
|  | 193-208 | FNRKEKTTKTTIEELK |  | 591-603 | LGYRSFINKAGII |  | 298-304 | GSNLEKA |
|  | 209-223 | SLVDVIKYEGTLDSQ |  | 607-619 | IGIPKVDGLPLIY | Gudiv_517 | 35-48 | ISLINKTNFEQASL |
|  | 300-308 | NTCPIDSIMHTE |  | 636-649 | FAAGGGSSGSSVRN |  | 52-61 | LVDWHQDFNV |
|  | 362-372 | DETDLSDANEWVQEV |  | 677-691 | GYNYNGLYGKYNLPQ |  | 68-82 | VENKSTYSSFSKQKG |
|  | 420-430 | KNRKFMNRQLI |  | 697-705 | GGGKDQKSS |  | 83-97 | SNVINNQWADENVSL |
|  | 53-59 | EKKIKED |  | 715-729 | GENFKTNIFKESVKS |  | 98-112 | ITNESDFNRFFNIKN |
| gudiv_93 | 5--10 | KNANKK |  | 730-735 | VPEDFK |  | 113-130 | NKYALDLKNKLSNIDFNN |
|  | 20-35 | IANNKFFSVTSSKALV | gudiv_357 | 5--18 | TTVGAQGSLNNFVH |  | 148-162 | LYDLSGYFINDFSII |
|  | 36-51 | VNDKADVVFRNIDKTK |  | 57-63 | YYKVSWE |  | 167-181 | QLNLREYYKNKYFGT |
|  | 52-67 | TFKLEKVMVFDGLNSA |  | 121-127 | ILKKPAY |  | 182-188 | KTNQIVQ |
|  | 68-83 | ENNEVNIPSTLKQQEL |  | 137-145 | NKVIRQVSM |  | 199-210 | LINKDILNQQIV |
|  | 84-99 | KDRSELEKPAVFVEIK |  | 176-185 | AEATPESLNW | Gudiv_546 | 5-ago. | NQIK |
|  | 100-115 | KQDKKEEKVEDKLKEK |  | 189-203 | AMPAVFEGMKHYLTD |  | 15-20 | STSLVS |
|  | 116-131 | TDQIKPQDQKGDEELN |  | 204-217 | LLQPGSIIDPNTPY |  | 30-44 | SQRSIDPVTYQLYK |
|  | 132-147 | KQKQPKEDASKQENKK |  | 244-250 | LIKQIRI |  | 45-59 | TKDQQLVEIVFKSDQ |
|  | 148-163 | EEITQQDPQNQGPKVE |  | 275-281 | QFTFAGT |  | 60-74 | ISEKELANTSFDFQ |
|  | 164-178 | KTEPTKKDAEKTQAQA |  | 305-319 | RFRNRFILDSIFAFM |  | 75-89 | QTSPQLKTRASFKPT |
|  | 179-196 | IDKKEEGSSSEEKKIG |  | 320-334 | LPKSWRKKLTDIRQP |  | 90-100 | KVVLDSANNQI |
|  | 197-212 | EIKPSEPEPQAQPEEQ | gudiv_388 | 5--13 | KSKKWVNYG | gudiv_560 | 30-44 | AKKDEQPKTKTGSQQ |
|  | 213-228 | NTPKSEPLIKEQEEQK |  | 32-46 | KAKKEPIIQTKATEQ |  | 45-59 | QQEENKQIINQSKPK |
|  | 229-244 | NGQGQGQKDESSKTQE |  | 47-58 | TNENNQNNEEIA |  | 60-70 | ETKQEYNPNIF |
|  | 245-260 | ANNGGNTSEQENEKTK |  | 65-77 | AKKEAAKEKINNL |  | 81-91 | KEDNDEQLKTK |
|  | 261-276 | EKDPLENKESDPDPNT | gudiv_398 | 31-45 | NNSSSNKPITNQFND |  | 104-110 | DKYVAKK |
|  | 277-292 | QEITKQEDPETKKPTL |  | 55-69 | DINYNKIKVEDQQLI |  | 122-129 | DKLREDPT |
|  | 293-308 | SSDVQWSSLRVAHWNV |  | 70-78 | KQKVANLQQ |  | 141-155 | EPMFTRKAFDPEHLK |
|  | 309-324 | QHQSGKDQDKNEALAR |  | 83-97 | VNIINNKSKFEHYYN |  | 156-170 | TMVNTLESNKHWSID |
|  | 325-340 | IILKHKFDVVGLTEIM |  | 112-126 | IDLYNKATTKREPIK |  | 171-182 | QLRSEMDNRHEK |
|  | 341-356 | PVKDGMTSEEKEQAVK |  | 127-141 | LKEPTIIKKDIINNS |  | 190-204 | EELGNKTDFLDNIPD |
|  | 357-372 | RVVDLMNEFSNTTNYE |  | 182-189 | VSYGSKQS |  | 205-219 | RLGKYHREELPPKPT |
|  | 373-388 | YLISDNLEGRENKITE |  | 201-205 | DNKLK |  | 231-245 | ERAVKERETQREYFY |
|  | 389-404 | STFHTSTERIGVIYNK |  | 221-225 | ENYNI |  | 246-260 | MRKWTEEDPWHFYKE |
|  | 405-420 | EKVHPIPFANGNIGHI |  | 232-246 | VNETVDLNRIDLTPL |  | 261-265 | NGISK |
|  | 421-436 | YSNKPGHGIWTQKDVD |  | 247-253 | VNKDNEK |  | 278-292 | GSDKWNTKFKESNHN |
|  | 437-452 | YSRPPFSVKMQSVGKV |  | 268-282 | YKLFDSDYSDKSYWD |  | 302-312 | YEKTLTKDQRQ |
|  | 453-468 | TSPGYRKGKKQEKNYS |  | 283-297 | NFEIINTHTELEDKH |  | 331-336 | MSSGEQ |
|  | 469-484 | KKALRDLGVERILKDI |  | 332-346 | RIQTSNDGINFKDVV |  | 352-366 | NLNLHQIQEKTENNK |
|  | 485-500 | EKPGKFLENDQGSREL |  | 347-356 | NQDVISTTSW |  | 367-381 | TVSKEEQYIEKLSTT |
|  | 501-516 | DDAEKDEDKLGNEHKE |  | 358-369 | NLAVNDDNTSDW |  | 406-411 | AATHTN |
|  | 517-532 | GFENLLKDDYEHKTSL |  | 376-380 | TAKAK |  | 421-428 | THLKTPTN |
|  | 533-548 | STITNKMANPYDKIFK |  | 409-421 | YGVDKQTKRESMY |  | 456-461 | SRIQRR |
|  | 549-564 | NSNLETRDAKLFNKVF |  | 425-439 | DDTIKSFSFNDQVVN |  | 463-475 | KTAFESTNKEKKI |
|  | 565-580 | EEQILNDEWYQKVKKT |  | 440-454 | ATDIYDFKRKQYIIN |  | 484-498 | KHTEQNDVILPKVIT |
|  | 581-596 | KGSKWPKTEPKEKAAK |  | 455-463 | SNHNLDINK |  | 499-510 | DEWQLNRDYKHI |
| gudiv_103 | 29-43 | CSKSKSNLELEAIYE |  | 486-495 | ETYLKDETNK |  | 514-519 | LLNPKS |
|  | 44-49 | KKDQNL |  | 507-511 | DQNNK | gudiv_633 | 27-41 | SQKNKNPSTKSTPKK |
|  | 68-82 | YNNETKAFNFDNNTD |  | 523-537 | KNNLNSNDQLTDKLE |  | 42-56 | SLLEENIKITDPTVV |
|  | 83-97 | LVEQKDHIELINSKA |  | 538-545 | LSSNTVVN |  | 57-71 | KQTQPLVALKPAQPI |
|  | 98-112 | ALDQYLENRVVPNLN |  | 558-569 | NNYSYKSDQTYQ |  | 72-79 | TTSFLSQP |
|  | 113-127 | QASLSDLIKDHFKDF |  | 578-585 | LDYASINK |  | 88-94 | NNYLELK |
|  | 128-132 | DFSQQ |  | 608-622 | LDNLTFRNEDKNKQK |  | 105-111 | NSLYKNK |
|  | 141-155 | RDSLDLSNLKLESGW |  | 634-645 | NNEFIEYDQLKNI |  | 131-136 | KTKIAN |
|  | 156-168 | NISSFRQSTNRFK |  | 671-683 | NNEKLISKETKE |  | 164-178 | LNNDSKSSDYQITNE |

**Additional table 1.** Sequences of the most significant B lymphocyte epitopes (highest thresholds in the Pepipred 2.0 predictor) accompanied by the position of the respective epitope in each UdLAMP.

**Continuation**

| **LAMPs** | **Position** | **Epitopes** | **LAMPs** | **Position** | **Epitopes** | **LAMPs** | **Position** | **Epitopes** |
| --- | --- | --- | --- | --- | --- | --- | --- | --- |
| gudiv_103 | 173-186 | FDQKDETQSTNKVV | gudiv_398 | 741-745 | IKDYS | gudiv_633 | 179-193 | KEDLSVFSKLSDIKL |
|  | 197-211 | KLSSFRFARVDLVGS |  | 773-783 | SRIQNDYYGAN |  | 209-220 | LRLNNLSKWKDK |
|  | 212-217 | KTTSST |  | 797-805 | EIFDKKLKS |  | 229-243 | LNTNEEFYSLETKIK |
| gudiv_159 | 19-33 | NDKTPNPSKNKETNT |  | 818-831 | NWLSSSNDSNSIKD |  | 344-249 | SPTQDF |
|  | 34-48 | QQSSSSTPKIMTGQE |  | 839-853 | KEFNETKIVNDNLAR |  | 257-265 | VYDQYKIVK |
|  | 49-63 | DKMKPKAQAKQTNKQ |  | 865-870 | VASLNN |  | 272-286 | VYLDEDERKRTKYTS |
|  | 64-78 | TNNNPNTSHNHNNST |  | 882-889 | TDYLDFEV |  | 287-301 | IPLDDSITNKILSVK |
|  | 79-93 | MEPNSKSGDEIKNGS |  | 982-900 | VKLNQTYDQ |  | 302-316 | YKTNLNWNPFTNDKN |
|  | 94-108 | KTDENKNNLGNSTTN |  | 922-936 | RFSLDNEKILKKINE |  | 327-332 | NYLTTD |
|  | 109-121 | DSTKKQNANPPVV |  | 937-946 | LKQTSSLDQY |  | 343-352 | HFENEKNIHH |
| gudiv_162 | 32-46 | NEKKDDNPQLNQQSQ |  | 958-971 | NENEFNNQFDLSKS |  | 358-367 | RALDNNHEDA |
|  | 47-61 | TNSVTTPLLDPKDTS |  | 984-998 | NKPTIRRKQKYVNES |  | 378-392 | EELATKIGFDSLDKN |
|  | 62-70 | KESYKPSNE |  | 999-1011 | FEYRIKQTPTIKD |  | 393-399 | IKTDFNK |
|  | 73-87 | AKISKAANDLNVTFT |  | 1030-1044 | KELITDKNVVFQSLE |  | 409-423 | EEHSYYNNDYYNTYI |
|  | 88-102 | QLANRPNLKSSDIMF |  | 1045-1059 | ATEKDDDLIDLNQAK |  | 424-432 | AKKYDVDPK |
|  | 103-117 | DQIEQESIKFEIKGN |  | 1060-1064 | EILAD |  | 442-456 | YNYLPKFSSTKEIIK |
|  | 132-146 | DLDEHNNNVSFRTG |  | 1075-1089 | KKGLNQSHQDFYPDD |  | 457-462 | RRNSEP |
|  | 155-160 | HIPTST |  | 1099-1108 | DLPKFGKTI |  | 478-492 | QLDDIEQYKLKLKTK |
|  | 169-183 | DDLKSNPANANSKGQ |  | 1111-1124 | KQLIRAYDSGNEFG |  | 493-507 | KTDYETWYKKPKTDW |
|  | 184-198 | IQHIARAPSKSEVNN |  | 1138-1152 | IDENNQVVQKWVDNA | gudiv_635 | 31-45 | NENKNPATKSTPKKS |
|  | 199-213 | YLTTYTQLQRFEKDN |  | 1153-1167 | FSDSFSVNHFKRYQY |  | 46-60 | LLEENVKISDPTVVK |
|  | 214-228 | NPYVESLRRYRATSQ |  | 1168-1182 | HDFIKPINNQYLTED |  | 61-68 | QTQPLVAL |
|  | 229-243 | GLPADAPLSDVIKNV |  | 1183-1197 | DFKAQNEFDPNLKKE |  | 85-90 | LKFVDN |
|  | 244-258 | KVTENQKAEFDKKAK |  | 1198-1207 | IDLLDNSDVD |  | 101-106 | SYINKY |
|  | 259-269 | ALNLDTYDNQA |  | 1211-1225 | VVGRAESSSKTTLYR |  | 124-138 | NKTKIVNDGSAQVSF |
|  | 280-294 | ETGKVIGLNIQDNPG |  | 1226-1236 | NLNIDEFLNQK |  | 139-145 | ENLEDDN |
|  | 295-309 | QQASWVDYYNRLAYR |  | 1250-1264 | PKDKEVVIGTQLTNK |  | 158-172 | NNSNPIERQIINDQQ |
|  | 310-325 | SSGLARTLTNQTYKDI |  | 1265-1279 | LYQPGEYVSSRSGHI |  | 173-187 | DLSVFSKLSDIKLST |
|  | 336-350 | NQDPEHPHDELKSV |  | 1280-1294 | PRDDIDRWSSANNKL |  | 203-212 | LNNLLKWKDK |
|  | 362-367 | KPTNGS |  | 1307-1311 | KKLGH |  | 222-226 | NTNEK |
|  | 386-400 | TDETKNLSLTRINND |  | 1325-1336 | DKKQRYSPNKEF |  | 236-241 | SLNQDF |
|  | 401-415 | VGIGKTLRIDGLDDN |  | 1342-1348 | VNDYEQN |  | 249-257 | VYDQYKIVK |
|  | 416-428 | FSTFIFDMKKMKK |  | 1359-1364 | IHYNIK |  | 264-278 | VYLDEHERKWTKYTS |
|  | 440-454 | LNKDPSDYLVEEQKQ |  | 1370-1375 | IATKTV |  | 279-288 | IPLDDSITNQ |
|  | 455-461 | KYKDYKE |  | 1379-1386 | STDLKELD |  | 294-308 | YKTNLNWNPFTNDKD |
|  | 474-488 | GVEENALDYGRNPLS |  | 1393-1403 | NSEANNDLYIN |  | 335-342 | HFKNEQNL |
|  | 489-599 | NFPREDLKDGE |  | 1407-1421 | ITYKFSLLKSYFRI |  | 345-359 | KIISSRGLNNNYEDP |
|  | 511-525 | EKPEEKKIKFLKESY |  | 1422-1429 | GEIKQYGK |  | 370-375 | EELATK |
|  | 526-540 | LKNYKKIDVPLADLS |  | 1440-1446 | TFDRKWV |  | 378-391 | HDLIEKNIKTDFNK |
|  | 541-548 | KDANKDKY |  | 1457-1471 | FKNSDNSLVEQLHYD |  | 402-416 | KHSYYDRDYYNTFIA |
|  | 559-573 | SVGDYFLKQYVDDDQ |  | 1472-1486 | NQKLTTLYYDNKNVK |  | 417-424 | KKYDVDPK |
|  | 574-588 | KLVAKWHRSLWINQN |  | 1487-1501 | RIRELEPYIDDAEWS |  | 434-448 | YNYIPRFSSTKEIIK |
|  | 589-603 | EKFYEKISVNELTNE |  | 1502-1506 | LKSDY |  | 449-454 | RPDTEP |
|  | 604-618 | STFSKEELERGNI |  | 1517-1531 | IKHTLLSNNAFDREL |  | 470-484 | KLENIEEYKLKIDTK |
|  | 622-634 | GLRTFIDKPGLSD |  | 1547-1554 | AERITSKN |  | 485-499 | KTDYETWSKKPKTDW |
|  | 637-651 | IGVPFITKEPLETKVTD |  | 1556-1586 | SFIENVDNKPI | gudiv_663 | 59-73 | GANAVHELAFQNQNL |
|  | 652-662 | TDDQNNEKSVK |  | 1613-1627 | FRIKLGYPQNERIKD |  | 74-85 | LNQQYFRSGDLS |
|  | 674-688 | HYEPTGGASGSSIRN |  | 1652-1666 | NQIQNESDFGLGKTN |  | 89-103 | IKDNKRVDKNNKEID |
|  | 716-730 | GFNYNGLYGDYNLPQ |  | 1667-1678 | LYEYNLANTSAT |  | 119-129 | TKDTDPKKINP |
|  | 737-747 | GGQDQKKDKYG |  | 1689-1703 | SYYANKIYNPNQNVY |  | 145-159 | NGLSYHPLSTDKDTK |
|  | 757-771 | YPNSNMKTAFPEGVD |  | 1704-1712 | WKHNDGFIS |  | 160-174 | TSTLPINFETLNTQT |
|  | 772-786 | HEYQEFKFTELAKPS |  | 1716-1729 | WIRDDWGTVNREWD |  | 175-189 | NQYELNKTIYNEQKL |
|  | 787-796 | SSSSSSSSSS |  | 1744-1752 | YNRHPGSAG |  | 197-202 | DVYNKA |
| gudiv_164 | 30-44 | EDSKPIQEPKKDQQT |  | 1755-1762 | NKINDDPN |  | 228-242 | NVKDYNFRSQQDSEV |
|  | 45-59 | KQLNKSNDVNKEKIR |  | 1775-1788 | GEFYPNINANANAI |  | 272-286 | YEFIKGVKDLSIDLP |
|  | 73-87 | DLAKLSNDQRQQLKP |  | 1790-1800 | VDKATEPPVIG |  | 287-301 | QFGQPLTDQQIADRS |

**Additional table 1.** Sequences of the most significant B lymphocyte epitopes (highest thresholds in the Pepipred 2.0 predictor) accompanied by the position of the respective epitope in each UdLAMP.

**Continuation**

| **LAMPs** | **Position** | **Epitopes** | **LAMPs** | **Position** | **Epitopes** | **LAMPs** | **Position** | **Epitopes** |
| --- | --- | --- | --- | --- | --- | --- | --- | --- |
| gudiv_164 | 88-102 | KIDDLLKSENLSKLN | gudiv_398 | 1809-1823 | NKWDSELYKGYGYSV | gudiv_663 | 320-334 | NTSAKDGASFMLPEK |
|  | 103-108 | DPSFLD |  | 1824-1836 | YSWFNTNSSLNVR |  | 335-344 | NSADPLKNPL |
|  | 126-140 | HLDLEKLIKTTNNNN |  | 1848-1862 | NRANNNSPNSVDELV |  | 352-362 | SVDDKKVYKDG |
|  | 141-155 | NNNNNNSKEDKDKKD |  | 1863-1871 | DKDGKPSSS |  | 382-396 | ATLRVDRTNQELTSK |
|  | 156-170 | KKDNTTNKTDKVNNP |  | 1910-1920 | IKINQSSNRNH |  | 397-411 | NEALIKANNNNKELT |
|  | 171-185 | NQLNNENKENKQLES |  | 1947-1953 | RPEMTDT |  | 412-419 | QQQKEGAV |
|  | 186-200 | INKNQTTPESKPINK |  | 1957-1963 | FRQYPNY |  | 447-461 | KALFELENRKYTEDK |
|  | 201-212 | DNLKQQSNTNPN |  | 1990-2004 | AGANGSFSTGELFDS |  | 462-476 | EILDHLYYEHNNGIN |
|  | 225-236 | DNIVKNKYGGNK |  | 2015-2026 | DKRSPLELKAFK |  | 489-503 | PIVRDSFVSSRKFVE |
|  | 270-276 | NERKQLM |  | 2040-2048 | HPFKYAVPW |  | 504-518 | AIKKSVVSKKVDEKQ |
| gudiv_171 | 30-44 | KNLKQANIAFDKQTL | gudiv_402 | 31-45 | PNKSKTPEKPKQGTE |  | 532-547 | KATIGTDANNTAAAAN |
|  | 45-59 | NQHITNIKNLKTDQE |  | 46-60 | NGRGSGSMSGNNTGG |  | 557-571 | KVVASNQNVSDSWNE |
|  | 60-72 | LNNYYNKQMQGNI |  | 61-75 | TTSGSTGGNNGNGGS | gudiv_680 | 32-46 | NKSTQKSQIEKQEQK |
|  | 212-226 | PDFYKTSAFSEISDH |  | 76-90 | SKNNEQMNGLGTQPQ |  | 47-61 | LDQSQQSKTTTQSLS |
|  | 227-241 | LYPIIRSNLNFKSVA |  | 91-105 | NPKNNGGSSGSNTTP |  | 62-73 | IKDNSLVKNNNQ |
|  | 242-256 | DKLLLTDDARTRFIN |  | 106-115 | TTPSANKNAN |  | 96-110 | KEKDSNKFIRSNNRS |
|  | 153-162 | ISKNKTIDIN |  | 121-135 | PSGEESIALDPASDV |  | 111-117 | KIDNNKA |
|  | 175-187 | YKEDPENIEHVYK |  | 143-152 | NKLNPQQNLE |  | 122-130 | NNLDPNKSY |
|  | 198-202 | KKYPN |  | 163-177 | QNQQVKKAFAELKIW |  | 140-154 | SKTAVDGEQQYLNQD |
|  | 212-227 | IFNVLDQEGYIRRFFD |  | 206-217 | KNDRNFGEVSKD |  | 155-169 | IKDKEVNIKNEAEKS |
|  | 250-263 | NHLNNKNDFSFLNQ |  | 227-232 | NLATKQ |  | 170-184 | DTASSNSLNEEITTN |
|  | 286-300 | SLDYNNKALDMFDLN |  | 237-251 | VQSNKKVLTEEEKKK |  | 185-193 | KTESPKPKV |
|  | 301-305 | NFLVA |  | 270-283 | VPGQTDVGTPIKDK |  | 217-229 | LSENERLENIQPN |
|  | 310-324 | VVKRGDHKIGVNYKP |  | 294-308 | QDQWTTSTKLNTQIH |  | 253-267 | KNEQVYNYKFNKSDQ |
|  | 325-232 | VNMKHIDT |  | 309-318 | KIENNNIDIS |  | 268-273 | TTSWET |
|  | 338-345 | VELKKLKP |  | 322-329 | ISEFKEKK |  | 295-309 | TELVKHNTELQNQER |
| gudiv_179 | 9--23 | VVAACAPTKAKPAKP |  | 341-355 | DDNKAVTPSSQYRFD |  | 261-272 | PNEETVRGMNTV |
|  | 24-38 | TEKKVEQPQTSGTES |  | 356-362 | YEDPSAL |  | 382-389 | NQDPVDVK |
|  | 39-53 | SKTGSGSGNATTGTK | gudiv_410 | 28-42 | QEKEKPARKSTPKKS |  | 394-401 | ETLTKSGE |
|  | 54-68 | QGSENNTTTPSAGST |  | 43-57 | LLEENVKISDPTVVK |  | 410-424 | KDPNKQVDNIDEANL |
|  | 69-83 | NTNPGATQPTTPESG |  | 58-69 | QTRPLAALEPAK |  | 425-431 | LANPFKI |
|  | 84-98 | SKTKPEDSKGSGTDS |  | 96-101 | DSSYQD | gudiv_681 | 30-44 | STNPTLKSELNLEIV |
|  | 99-113 | ESKKDDKTENKPNLG |  | 110-124 | KPNNNDELINSNKAK |  | 45-57 | EKVLRPTNNQKYG |
|  | 114-128 | SDPSNGSDDTNKKTE |  | 125-131 | ISNDGSV |  | 62-76 | VNKAYADHFVQVDLV |
|  | 129-143 | TEGKVEEGKNDNTPK |  | 135-143 | FENLEDNNE |  | 77-91 | KSTTNTNSISSDKK |
|  | 144-158 | TAEENGTQSDTPTEP |  | 154-168 | EINSNNASNHQISNP |  | 92-95 | ISSSG |
|  | 159-173 | QAPPAANSGSDSTKK |  | 169-183 | KDDLSVFTKLSDIQL |  | 105-117 | IDLDSKYLIKK |
|  | 174-188 | DMEADPGKTDAKKME |  | 184-192 | ETNSQLIEY |  | 119-133 | FKKINDQNPLINQEY |
|  | 189-203 | GSGSEDPKAMGDTSG |  | 202-208 | NNLLKWK |  | 134-148 | KTKDLLNVHKVVNSS |
|  | 204-218 | SNNEQQNSELQLKTK |  | 220-227 | KTKERFES |  | 149-163 | TPTENLNQNNNPDQN |
|  | 219-233 | KDEAKASISKLSNLL |  | 251-265 | YKIVELKYFNEEYKD |  | 164-178 | PNPDQSLNQKGESKL |
|  | 234-248 | ANDKKEFEKQIDAIT |  | 266-272 | KKDNKDE |  | 179-193 | SSINKVFHDGIVYKK |
|  | 249-263 | KPEQNQINDILKEAT |  | 291-305 | YKTNLKANPFTNEKD |  | 194-208 | SNDSLEYKTAVDDEF |
|  | 264-278 | NKDTERKNKLTPTNS |  | 315-321 | LKYLSND |  | 220-234 | LDGGEPQYFNATAKD |
|  | 279-293 | VLNYDATNKRNYLRF |  | 331-343 | DRTDILDQNTEKY |  | 242-256 | SQLVSGSYQIKELKD |
|  | 294-308 | EFKTSKETFDKIKKQ |  | 345-357 | IKYVRETTPVEEY |  | 257-263 | KEADKQL |
|  | 309-323 | KLTLLLQVRGKDGSQ |  | 368-382 | EELSSTFKSSDLNKV | gudiv_759 | 19-28 | KQIKELQDEIQI |
|  | 324-338 | YNPVTDAAATWNDGS |  | 383-388 | VTTNFD |  | 34-48 | KTPNLSEDIVQQNKE |
|  | 360-370 | YFVKELNSPEG |  | 400-414 | YASTNFRREYRIKVP |  | 49-63 | RLANLEARLNFYKLN |
|  | 381-396 | ENDKKIELLNQPSQEI |  | 415-424 | KRVDINSQTK |  | 64-78 | LKPNVSLPKNSTVTE |
|  | 398-405 | VSYSTASA |  | 439-451 | FKTKKDVSKRPNW |  | 79-93 | IIDKFNNITNHFKDL |
| gudiv_180 | 29-43 | NNEQQQQQSVNQNQV | gudiv_412 | 170-184 | DDLSVFTKLSDIQLE |  | 94-81 | PADSHNLKASSNDN |
|  | 44-58 | LIKQENNTKNTKTKS |  | 185-196 | ANSQLTEYYYEE |  | 134-148 | EYLSDDESSEYENIR |
|  | 60-74 | LNKTSPISKEQPKVK |  | 202-207 | NNLLKW |  | 175-182 | IDPVNIDA |
|  | 75-89 | SETLVKKSFDSNNNN |  | 220-234 | KTKERFESLRSKIEN |  | 195-209 | LNLPDYDGSPAILRW |
|  | 90-104 | NNNNNNNNNSLEDNS |  | 251-265 | YKIVELKYFNEEYKD |  | 210-224 | DMKIHRNKTDKEIQD |

**Additional table 1.** Sequences of the most significant B lymphocyte epitopes (highest thresholds in the Pepipred 2.0 predictor) accompanied by the position of the respective epitope in each UdLAMP.

**Conclusion**

| **LAMPs** | **Position** | **Epitopes** | **LAMPs** | **Position** | **Epitopes** | **LAMPs** | **Position** | **Epitopes** |
| --- | --- | --- | --- | --- | --- | --- | --- | --- |
| gudiv_180 | 105-119 | KTKSETSVNKPTTPN | gudiv_412 | 266-272 | KKDNKDE | gudiv_759 | 225-239 | YENSDINQTEEGELL |
|  | 120-134 | QNNNNVVVTKPENSQ |  | 292-306 | KTNLKANPFTNEKDK |  | 240-252 | NDMGYIYKSMRN |
|  | 135-149 | PKQNNIQPIKVASDF |  | 316-321 | KYLSND |  | 269-283 | LDADEGYYGANSFQE |
|  | 150-164 | YPKQTQEPLAFSTLN |  | 332-346 | RADILDQNTKKNIIK |  | 284-298 | DEFKKKNGQAKYTHK |
|  | 165-179 | EQEIKARILSTDQST |  | 347-358 | YVSETTPVEEYN |  | 299-313 | KERQTKHANEENKPF |
|  | 180-190 | VSYYSHPDFRL |  | 368-382 | EELLSTFKSGALDID |  | 314-324 | SLTNEMLDDVY |
|  | 202-216 | DDSKRESTKLELLTK |  | 383-390 | NVVTTNFD |  | 269-283 | LDADEGYYGANSFQE |
|  | 217-231 | DNEPVDGVRWFIRTR |  | 403-417 | TSGYFRPEYRIKVPK |  | 284-288 | DEFKK |
|  | 232-246 | YPTDDVFSSEVNIKD |  | 418-426 | RVDINSQTK |  | 296-310 | KNGQAKYTHKKERQT |
|  | 261-266 | SYDGND |  | 434-448 | ENNEAPWFKTKKDVS |  | 331-345 | EENKPFSLTNEMLDD |
|  | 287-301 | YSDTETLNEFEIRES |  | 449-460 | KRSNTIPTANFQ |  | 356-367 | LKNKQEGNEPVE |
|  | 310-317 | KDWHHLSN |  | 472-486 | IDNLSEYNLKFDFKD |  | 373-387 | DQNNETEIDLSSLEQ |
|  | 334-345 | YEESEDPFSDQT |  | 487-498 | KEKDETIEWDSL |  | 397-403 | KNSDGTE |
|  | 407-421 | ARSASISLSNEQETT | gudiv_427 | 34-40 | GDNERDP |  | 414-423 | SITKKENAEN |
|  | 430-444 | EDFAKGRTFTNPYTK |  | 43-57 | SVKNTENNTKVIRFH |  | 434-442 | QIKEEDKIV |
|  | 445-459 | EEMGTKYRAAKMENF |  | 58-68 | PRDYQYLIGSK |  | 454-463 | VNLDNKYKET |
|  | 460-474 | TANDIDVKRVIHKTY |  | 78-92 | KMFNLFSYEMSQDEE |  | 470-484 | EEVYKDIDQFIEQKQ |
|  | 475-489 | TRKNEEVKWLTILTQ |  | 93-107 | FRTKYPHFFKGRNVV |  | 471-499 | KLYALQREIMKVDFS |
|  | 490-504 | WQYRDIEHLQKIVEQ |  | 108-115 | NRDQAYKE |  | 500-507 | ADPNSNDP |
|  | 505-511 | ETKGKID |  | 135-149 | FKKFRVGEFIGKHEY |  | 514-521 | TNDLSLAN |
|  | 513-527 | DGGHSNIEENFNQFR |  | 150-158 | YNTIKLKRE | - | - | - |
|  | 528-542 | FIVQNPLDKKEYQKA |  | 160-170 | INHVQKVDFSK | - | - | - |
