# Supplementary material for: Immunoinformatics and analysis of antigen distribution of Ureaplasma diversum strains isolated from different Brazilian states
Source: BMC Vet Res. 2020 Oct 7;16:379. doi: 10.1186/s12917-020-02602-1 (PMC7542862; doi:10.1186/s12917-020-02602-1)
Supplement: Supplementary file 2 — Additional file 2: Table S2. Sequence of the most representative CD8+ T lymphocyte epitopes and respective position in each UdLAMP predicted using NetBoLApan v1.0. Peptides with an 8-amino acid window of the BoLA-1 *02301, BoLA-3 *00201, BoLA-2 *01201 and BoLA-6 *01301 alleles are represented. [file 12917_2020_2602_MOESM2_ESM.docx]

**Additional table 2.** Sequence of the most representative CD8+ T lymphocyte epitopes and respective position in each UdLAMP predicted using NetBoLApan v1.0. Peptides with an 8-amino acid window of the BoLA-1 *02301, BoLA-3 *00201, BoLA-2 *01201 and BoLA-6 *01301 alleles are represented.

|  | **BoLA-1** ***02301** | | **BoLA-3** ***00201** | | **BoLA-2** ***01201** | | **BoLA-6** ***01301** | |
| --- | --- | --- | --- | --- | --- | --- | --- | --- |
| **LAMPs** | **Position** | **Epitopes** | **Position** | **Epitopes** | **Position** | **Epitopes** | **Position** | **Epitopes** |
| gudiv_61 | 357-366 | VMIGGNASY | - | - | 393-402 | KSKLWWEPK | - | - |
|  | - | - | - | - | 357-366 | VMIGGNASY | - | - |
| gudiv_66 | - | - | 124-133 | KTSSYQFTL | - | - | 344-353 | RINHINPKL |
|  | - | - | 240-249 | FAYINKIAL | - | - | - | - |
| gudiv_85 | - | - | 440-449 | YTVEIPSYL | 280-289 | ATIKNGILK | 4--13 | KIKYKWMSL |
|  | - | - | - | - | 336-345 | SVPQRGAGK | 149-158 | SQSLKATQI |
|  | - | - | - | - | 360-369 | STNTGDSSK | - | - |
|  | - | - | - | - | 414-423 | SSSINNTAK | - | - |
| gudiv_91 | 323-332 | RMMQIQQIH | 32-41 | WSIGLIIGL | - | - | 310-310 | MLITKNDTL |
|  |  |  | 92-101 | YTMTLGSNL | - | - | - | - |
| gudiv_93 | 52-61 | FKLEKVMVF | - | - | - | - | 110-119 | KLKEKTDQI |
|  | - | - | - | - | - | - | 325-334 | ILKHKFDVV |
| gudiv_103 | - | - | 57-66 | YNFELTNPL | 157-166 | SSFRQSTNR | 160-169 | RQSTNRFKI |
|  | - | - | 162-171 | STNRFKINL | 180-189 | STNKVVSTK | - | - |
|  | - | - | - | - | 188-197 | KTFFITINK | - | - |
|  | - | - | - | - | 211-220 | KTTSSTNSK | - | - |
| gudiv_159 | 112-121 | KQNANPPVV | - | - | 104-113 | STTNDSTKK | - | - |
| gudiv_162 | 86-95 | TQLANRPNL | 45-54 | QTNSVTTPL | 83-92 | VTFTQLANR | 86-95 | TQLANRPNL |
|  | 269-278 | VKAWTIPSY | 308-317 | RSSGLARTL | 139-148 | VSFRTGKAK | 304-313 | RLAYRSSGL |
|  | - | - | 693-702 | VSIFHSSFL | 314-323 | RTLTNQTYK | 774-783 | YQEFKFTEL |
|  | - | - | - | - | 416-425 | STFIFDMKK | - | - |
|  | - | - | - | - | 522-531 | ESYLKNYKK | - | - |
|  | - | - | - | - | 557-566 | SSVGDYFLK | - | - |
|  | - | - | - | - | 599-608 | LTNESTFSK | - | - |
| gudiv_164 | 59-68 | QQIDAFIKL | - | - | 20--29 | ASILIACSK | 116-125 | LQSFIKNQL |
|  | 204-213 | QQSNTNPNY | - | - | - | - | - | - |
| gudiv_171 | 2--11 | KQANKIVLI | 74-83 | LTAMFNSDL | 138-147 | SSFFLNDKK | 2--11 | KQANKIVLI |
|  | 65-74 | KQMQGNISL | 235-244 | STIPFENLL | - | - | 65--74 | KQMQGNISL |
|  | - | - | - | - | - | - | 207-216 | KLHPIFNVL |
| gudiv_179 | - | - | - | - | 288-297 | RNYLRFEFK | - | - |
|  | - | - | - | - | 376-385 | LSYMENDKK | - | - |
| gudiv_180 | 151-160 | KQTQEPLAF | 8--17 | ISALLCSAV | 143-152 | KVASDFYPK | 188-197 | RLKTNYVIL |
|  | 383-392 | HQYHIWNVV | 359-368 | YSKGYKMLL | 343-352 | QTAYSALVK | 383-392 | HQYHIWNVV |
|  | 489-498 | WQYRDIEHL | 653-662 | SSMQYRTTI | 353-362 | ASVCTGYSK | 489-498 | WQYRDIEHL |
|  | - | - | - | - | 419-428 | TTYNYFLIR | 755-764 | RIKPNGTQL |
|  | - | - | - | - | 435-444 | RTFTNPYTK | - | - |
|  | - | - | - | - | 553-562 | SSNYIIKIK | - | - |
|  | - | - | - | - | 678-687 | GTLGIWVRK | - | - |
| gudiv_228 | 50-59 | KQFSDSSVI | - | - | 97-106 | RMNKINSFK | 50-59 | KQFSDSSVI |
|  | 96-105 | ARMNKINSF | - | - | - | - | 190-199 | KSIHHNGIL |
|  | 119-128 | KEFNHRFIF | - | - | - | - | - | - |
|  | - | - | - | - | 280-289 | TSFFTSDYK | - | - |
|  | - | - | - | - | 322-331 | AIADTILTK | - | - |

**Additional table 2.** Sequence of the most representative CD8+ T lymphocyte epitopes and respective position in each UdLAMP predicted using NetBoLApan v1.0. Peptides with an 8-amino acid window of the BoLA-1 *02301, BoLA-3 *00201, BoLA-2 *01201 and BoLA-6 *01301 alleles are represented.

|  | **BoLA-1** ***02301** | | **BoLA-3** ***00201** | | **BoLA-2** ***01201** | | **BoLA-6** ***01301** | |
| --- | --- | --- | --- | --- | --- | --- | --- | --- |
| **LAMPs** | **Position** | **Epitopes** | **Position** | **Epitopes** | **Position** | **Epitopes** | **Position** | **Epitopes** |
| gudiv_287 | 65-74 | LQSAVQPVF | 180-189 | FSAFENKTL | 67-76 | SAVQPVFFK | 3--12 | SLFKKELAI |
|  | - | - | 327-336 | ISVNFFRPI | - | - | 316-325 | GQKYKDIAL |
|  | - | - | 561-570 | WTNSFYILL | - | - | - | - |
|  | - | - | 624-633 | WNMALEYSL | - | - | - | - |
| gudiv_331 | - | - | - | - | 41-50 | KTMTEQKEK | - | - |
|  | - | - | - | - | 51-60 | NSMDSAANK | - | - |
|  | - | - | - | - | 55-64 | SAANKQTNK | - | - |
| gudiv_357 | 257-266 | FMFYGIIRF | 111-120 | IAAAIFFPL | 114-123 | AIFFPLILK | - | - |
|  | 273-282 | SQFTFAGTY | 177-186 | ATPESLNWL | 313-322 | SIFAFMLPK | - | - |
| gudiv_398 | 40-49 | NQFNDAMNL | 38-47 | ITNQFNDAM | 23-32 | TSLAIACSK | 654-663 | KQSEAISSL |
|  | 412-421 | KQTKRESMY | 42-51 | FNDAMNLSL | 26-35 | AIACSKAKK | 869-878 | NQTAKIMLL |
|  | 567-576 | YQYLKNLHH | 391-400 | YYAGWRNPL | 189-198 | SVLINWFTK | 928-937 | KILKKINEL |
|  | 654-663 | KQSEAISSL | 700-709 | SNFSYYKAL | 334-343 | TSNDGINFK | 990-999 | KQKYVNESF |
|  | 908-917 | HKYNPALVL | 783-792 | KTFAFYQLL | 369-378 | LSIKFNTAK | 1424-1433 | KQYGKNKAF |
|  | 1114-1123 | RAYDSGNEF | 906-915 | ITHKYNPAL | 469-478 | VSFNNPMIK | 1430-1439 | KAFVRFEVL |
|  | 1185-1194 | AQNEFDPNL | 1406-1415 | ITYKNFSLL | 659-668 | ISSLKAIVK | 1557-1557 | KLKLDFEQL |
|  | 1424-1433 | KQYGKNKAF | 1868-1877 | SSSSFDLQL | 702-711 | FSYYKALTK | 1957-1966 | RQYPNYSPI |
|  | 1533-1542 | EMYAGMLVF | 1896-1905 | ILMKFRNYL | 706-715 | KALTKTIIK | - | - |
|  | 1957-1966 | RQYPNYSPI | - | - | 722-731 | ASFLSDEIK | - | - |
|  | - | - | - | - | 785-794 | FAFYQLLNK | - | - |
|  | - | - | - | - | 821-830 | SSNDSNSIK | - | - |
|  | - | - | - | - | 865-874 | ASLNNQTAK | - | - |
|  | - | - | - | - | 1154-1163 | DSFSVNHFK | - | - |
|  | - | - | - | - | 1216-1225 | SSSKTTLYR | - | - |
|  | - | - | - | - | 1287-1296 | SSANNKLIK | - | - |
|  | - | - | - | - | 1313-1322 | ISFRLGFVK | - | - |
|  | - | - | - | - | 1544-1553 | QVAERITSK | - | - |
|  | - | - | - | - | 1882-1891 | KTSFDHAKK | - | - |
|  |  |  |  |  | 2043-2052 | YAVPWFYKK | - | - |
| gudiv_402 | 2--11 | RKINKKLIL | 111-120 | KNANFSISL | 26--35 | ASCTPNKSK | 360-369 | ALFTKNDSL |
|  | 271-280 | GQTDVGTPI | - | - | 182-191 | ITLLFVGLK | - | - |
| gudiv_410 | 56-65 | KQTRPLAAL | - | - | 423-432 | KTINVELEK | 56-65 | KQTRPLAAL |
|  | 180-189 | IQLETNSQL | - | - | 434-443 | RALWFKTKK | 180-189 | IQLETNSQL |
| gudiv_412 | 56-65 | KQTRPLVAL | - | - | - | - | 56-65 | KQTRPLVAL |
|  | 180-189 | IQLEANSQL | - | - | - | - | 180-189 | IQLEANSQL |
|  | - | - | - | - | - | - | 226-235 | SLRSKIENI |
|  | - | - | - | - | - | - | 458-467 | FQNYPIFLL |
| gudiv_427 |  |  | - | - | 93-102 | RTKYPHFFK | - | - |
| gudiv_442 | - | - | - | - | 87-96 | KTYEKQLRK | 1--10 | KKYQKVLLL |
|  | - | - | - | - | 166-175 | YSSYFLVNK | 53-62 | DQRTRFTHL |
|  | - | - | - | - | - | - | 71-80 | KQNQKEAVF |
|  | - | - | - | - | - | - | 154-163 | KQVNVDQFL |
|  | - | - | - | - | - | - | 211-220 | QQMIKYKKL |

**Continuation**

**Additional table 2.** Sequence of the most representative CD8+ T lymphocyte epitopes and respective position in each UdLAMP predicted using NetBoLApan v1.0. Peptides with an 8-amino acid window of the BoLA-1 *02301, BoLA-3 *00201, BoLA-2 *01201 and BoLA-6 *01301 alleles are represented.

**Conclusion**

|  | **BoLA-1** ***02301** | | **BoLA-3** ***00201** | | **BoLA-2** ***01201** | | **BoLA-6** ***01301** | |
| --- | --- | --- | --- | --- | --- | --- | --- | --- |
| **LAMPs** | **Position** | **Epitopes** | **Position** | **Epitopes** | **Position** | **Epitopes** | **Position** | **Epitopes** |
| gudiv_457 | 44-53 | FMIQPKHEL | 138-147 | FSFVHTYPL | - | - | 44-53 | FMIQPKHEL |
|  | 161-170 | RKYTEVSQF | - | - | - | - | 138-147 | FSFVHTYPL |
| gudiv_458 | 488-497 | KKYKATKVF | 283-292 | IAYALNPTL | 233-242 | KTINIIENK | 192-201 | TLYFNKSQL |
|  | - | - | 357-366 | YTNQINQPV | 269-278 | LAMFKVGFK | 221-230 | KLINQSDQL |
|  | - | - | - | - | 477-486 | SLFESIFNK | - | - |
|  | - | - | - | - | 481-490 | SIFNKRSKK | - | - |
|  | - | - | - | - | 494-503 | KVFFVVVNK | - | - |
| gudiv_499 | 7--16 | KQILISTSL | 17-26 | TTFGLTSLL | 294-303 | LSVGSNLEK | 7--16 | KQILISTSL |
|  | 36-45 | SKYEQIPTL | - | - | - | - | 121-130 | IQKANQTYL |
|  | 236-245 | TQYHVAPTI | - | - | - | - | 236-245 | TQYHVAPTI |
| gudiv_517 | - | - | 139-148 | STIFSKAAL | 70-79 | KSTYSSFSK | - | - |
|  | - | - | - | - | 188-197 | RSYYLVVNK | - | - |
| gudiv_546 | 120-129 | SKYFKSFQL | - | - | 37-46 | VTYQLYKTK | 120-129 | SKYFKSFQL |
|  | - | - | - | - | 55-64 | KSDQKISEK | - | - |
| gudiv_560 | 322-331 | FMNQSSLLM | 320-329 | YNFMNQSSL | 20--29 | SVLVASCSK | 310-319 | RQYWDMDFI |
|  | 334-343 | EQYDLYKAY | 379-388 | TTVEQLLAL | 146-155 | KAFDPEHLK | - | - |
|  | 395-404 | LQANHILGF | - | - | 385-394 | LALERLALK | - | - |
|  | 457-466 | IQRRFKTAF | - | - | 462-471 | KTAFESTNK | - | - |
| gudiv_633 | 56-65 | KQTQPLVAL | - | - | 212-221 | TTMTDELTK | 56-65 | KQTQPLVAL |
|  | - | - | - | - | 299-308 | RSLHNVFVR | - | - |
|  | - | - | - | - | 387-396 | RTNQELTSK | - | - |
| gudiv_635 | 59-68 | KQTQPLVAL | 15--24 | VASSISIPL | 284-293 | ITNQTLSVK | 59-68 | KQTQPLVAL |
|  | 270-279 | RKWTKYTSI |  |  | 328-337 | YAHFPTHFK | 228-237 | SLRIKIKSL |
| gudiv_663 | 75-84 | QQYFRSGDL | 15--24 | TSALVIVPL | 212-221 | TTMTDELTK | 75-84 | QQYFRSGDL |
|  | 174-183 | NQYELNKTI | 335-344 | SADPLKNPL | 299-308 | RSLHNVFVR | 305-314 | FVRVKNIKL |
|  | 516-525 | KQQYANLAF | 519-528 | YANLAFAAL | 387-396 | RTNQELTSK | - | - |
| gudiv_680 | - | - | 8--17 | LMMVFLASL | 33-42 | STQKSQIEK | - | - |
|  | - | - | - | - | 54-63 | TTTQSLSIK | - | - |
|  | - | - | - | - | 181-190 | TTNKTESPK | - | - |
|  | - | - | - | - | 261-270 | ISTLFDKYK | - | - |
|  | - | - | - | - | 401-410 | YTITHVYIK | - | - |
| gudiv_681 | - | - | - | - | 192-201 | KSNDSLEYK | - | - |
|  | - | - | - | - | 246-255 | GSYQIKELK | - | - |
| gudiv_759 | 113-122 | GQFAAGLSY | 121-130 | YNGDISFAL | 409-418 | NSIKSITKK | 48-57 | RLANLEARL |
|  | - | - | 316-325 | TLAELVMHL | 465-474 | KTLNEEVYK | 55-64 | RLNFYKLNL |
|  | - | - | - | - | - | - | 259-268 | VLRSIYDHL |
|  | - | - | - | - | - | - | 480-489 | EQKQKLYAL |
|  | - | - | - | - | - | - | 484-493 | KLYALQREI |

- Absence of epitopes
