# Supplementary material for: Immunoinformatics and analysis of antigen distribution of Ureaplasma diversum strains isolated from different Brazilian states
Source: BMC Vet Res. 2020 Oct 7;16:379. doi: 10.1186/s12917-020-02602-1 (PMC7542862; doi:10.1186/s12917-020-02602-1)
Supplement: Supplementary file 3 — Additional file 3: Table S3. Sequence of the most representative CD8+ T lymphocyte epitopes and respective position in each UdLAMP predicted using NetBoLApan v1.0. Peptides with an 8-amino acid window of the BoLA-3 *00101, BoLA-4 *04101, BoLA-T2C, and BoLA-T5 alleles are represented. [file 12917_2020_2602_MOESM3_ESM.docx]

**Additional table 3:** Sequence of the most representative CD8+ T lymphocyte epitopes and respective position in each UdLAMP predicted using NetBoLApan v1.0. Peptides with an 8-amino acid window of the BoLA-3 *00101, BoLA-4 *04101, BoLA-T2C, and BoLA-T5 alleles are represented**.**

| **LAMPs** | **BoLA-3 *00101** | | **BoLA-4 *04101** | | **BoLA-T2C** | | **BoLA-T5** | |
| --- | --- | --- | --- | --- | --- | --- | --- | --- |
|  | Position | Epitopes | Position | Epitopes | Position | Epitopes | Position | Epitopes |
| gudiv_61 | 64-73 | AGAAWFSSL | - | - | - | - | 357-366 | VMIGGNASY |
| gudiv_66 | 154-163 | YTPTKDNSL | - | - | - | - | 88-97 | RLKRSFYLY |
|  | 348-357 | INPKLSSII | - | - | - | - | 91-100 | RSFYLYYSF |
|  | - | - | - | - | - | - | 126-135 | SSYQFTLQF |
|  | - | - | - | - | - | - | 128-137 | YQFTLQFKV |
|  | - | - | - | - | - | - | 361-370 | ILFDPYYYF |
| gudiv_85 | 98-107 | TNNSLISDL | 164-173 | EEHDHSSHM | 140-149 | DTNPTNVEL | 127-136 | YSLKKVSVY |
|  | 144-153 | TNVELSQSL | 479-488 | SEDLSSKGL | - | - | - | - |
| gudiv_91 | 32-41 | WSIGLIIGL | - | - | 88-97 | MINNYTMTL | 86-95 | YKMINNYTM |
|  | 92-101 | YTMTLGSNL | - | - | 211-220 | DVIKYEGTL | 116-125 | LLFSGVVRY |
|  | 150-159 | FGEFLPKNF | - | - | 310-319 | MLITKNDTL | - | - |
|  | - | - | - | - | 376-385 | EVNEYTWKL | - | - |
|  | - | - | - | - | 409-418 | SMGKYVSKL | - | - |
| gudiv_93 | - | - | 86-95 | SELEKPAVF | - | - | 52-61 | FKLEKVMVF |
|  | - | - | 172-181 | EEKTQAQAI | - | - | - | - |
|  | - | - | 189-198 | SEEKKIGEI | - | - | - | - |
|  | - | - | 370-379 | YEYLISDNL | - | - | - | - |
|  | - | - | 546-555 | NEHFAFKSL | - | - | - | - |
| gudiv_103 | 57-66 | YNFELTNPL | 84-93 | EQKDHIELI | 90-99 | ELINSKAAL | 164-173 | NRFKINLVF |
| gudiv_162 | 308-317 | RSSGLARTL | 459-468 | KEYADFAVI | 80-89 | DLNVTFTQL | 269-278 | VKAWTIPSY |
|  | - | - | 608-617 | EELERGNIM | 614-623 | NIMSYQIGL | 294-303 | QQASWVDYY |
|  | - | - | 713-722 | SEGFNYNGL | - | - | 299-308 | VDYYNRLAY |
| gudiv_164 | 231-240 | YGGNKAFAF | - | - | - | - | - | - |
| gudiv_171 | - | - | - | - | 164-173 | NLVFVGSSL | 65-74 | KQMQGNISL |
|  | - | - | - | - | 243-252 | LLVDYYNHL | 204-213 | YIFKLHPIF |
|  | - | - | - | - | 294-303 | DMFDLNNFL | - | - |
| gudiv_179 | - | - | 250-259 | EQINQINDI | 272-281 | KLTPTNSVL | - | - |
| gudiv_180 | - | - | 164-173 | EQEIKARIL | 242-251 | NIKDSVITL | - | - |
|  | - | - | 478-487 | EEVKWLTIL | 313-322 | HLSNFQKAL | - | - |
|  | - | - | 672-681 | VELNDIGTL | - | - | - | - |
| gudiv_228 | - | - | 119-128 | KEFNHRFIF | - | - | 64-73 | TMFFMFFSF |
|  | - | - | 147-156 | KEMNVIMVI | - | - | 96-105 | ARMNKINSF |
|  | - | - | - | - | - | - | 119-128 | KEFNHRFIF |
| gudiv_287 | 582-591 | LGNLLSKHL | 208-217 | YEFENDFYL | 341-350 | TVSGTMWIL | 65-74 | LQSAVQPVF |
|  | 606-615 | IGIPKVDGL | 316-325 | GQKYKDIAL | 378-387 | DLVGISFTF | 622-631 | HYWNMALEY |
|  | 624-633 | WNMALEYSL | 674-683 | SEGYNYNGL | 535-544 | SLYTVGYPV | - | - |
|  | - | - | - | - | 664-673 | ALVAVAAAL | - | - |
| gudiv_331 | - | - | 45-54 | EQKEKMNSM |  |  | - | - |
| gudiv_357 | - | - | - | - | 76-85 | ILCARLWSL | 57-66 | YKVSWEPAF |
|  | - | - | - | - | - | - | 257-266 | FMFYGIIRF |
|  | - | - | - | - | - | - | 273-282 | SQFTFAGTY |
| gudiv_388 | 11--20 | YGFGLVALV | 54-63 | EEIALMQAF | 17--26 | ALVGLSTSL | - | - |

**Additional file 3:** Sequence of the most representative CD8+ T lymphocyte epitopes and respective position in each UdLAMP predicted using NetBoLApan v1.0. Peptides with an 8-amino acid window of the BoLA-3 *00101, BoLA-4 *04101, BoLA-T2C, and BoLA-T5 alleles are represented

**Continuation**

| **UdLAMPs** | **BoLA-3 *00101** | | **BoLA-4 *04101** | | **BoLA-T2C** | | **BoLA-T5** | |
| --- | --- | --- | --- | --- | --- | --- | --- | --- |
|  | Position | Epitopes | Position | Epitopes | Position | Epitopes | Position | Epitopes |
| gudiv_398 | 42-51 | FNDAMNLSL | 310-319 | NEIGFKFFL | 15-24 | SLSTIIGSL | 412-321 | KQTKRESMY |
|  | 183-192 | YGSKQSSVL | 634-643 | NEFIEYDQL | 97-106 | ELKTLYQTL | 559-568 | YSYKSDQTY |
|  | 700-709 | SNFSYYKAL | 681-690 | KEKLLLTLL | 210-219 | IIYGAFSFL | 567-546 | YQYLKNLHH |
|  | 779-788 | YGANKTFAF | 869-878 | NQTAKIMLL | 284-293 | EIINTHTEL | 654-663 | KQSEAISSL |
|  | 1256-1265 | IGTQLTNKL | 959-968 | NEFNNQFDL | 317-326 | FLGTYAKTL | 859-868 | YRFMNVASL |
|  | 1329-1338 | YSPNKEFVI | 1214-1223 | AESSSKTTL | 487-496 | YLKDETNKL | 908-917 | HKYNPALVL |
|  | 1818-1827 | YGYSVYSWF | 1382-1391 | KELDKHITL | 511-520 | LINKYLVAL | 1019-1028 | FLFNNNVIF |
|  | - | - | 1513-1522 | SKYIKHTLL | 595-604 | DLVNEANFL | 1352-1361 | VMLNNLIHY |
|  | - | - | 1957-1966 | RQYPNYSPI | 623-632 | NLVAKITKL | 1424-1433 | KQYGKNKAF |
|  | - | - | - | - | 738-747 | NLIKDYSAL | 1533-1542 | EMYAGMLVF |
|  | - | - | - | - | 804-813 | SVTKANYFL | 1568-1577 | SSFIYSSSF |
|  | - | - | - | - | 1356-1365 | NLIHYNIKV | 1587-1596 | INYKLTITY |
|  | - | - | - | - | 1405-1414 | SITYKNFSL | 1682-1691 | LLYNDVSYY |
|  | - | - | - | - | 1444-1453 | WVLGKNWYL | 1957-1966 | RQYPNYSPI |
|  | - | - | - | - | 1531-1540 | DLEMYAGML | - | - |
|  | - | - | - | - | 1896-1905 | ILMKFRNYL | - | - |
|  | - | - | - | - | 1920-1929 | HIVPVLNPL | - | - |
| gudiv_402 | 242-251 | VNYDLYEHL | 177-186 | KENEPITLL | 9--18 | ILFSSLITL | - | - |
|  | 284-293 | NGILLNDTL | - | - | 360-369 | ALFTKNDSL | - | - |
| gudiv_410 | - | - | 56-65 | KQTRPLAAL | 12--21 | SLFLVSIPV | 90-99 | LSFKLDSSY |
|  | - | - | 354-363 | EEYNKKFFI | 82-91 | KLVDSKYTL | 242-351 | YLYSVYDQY |
| gudiv_412 | - | - | 354-363 | EEYNKKFFI | 12--21 | SLFLVSIPV | 90-99 | LSFKLDSSY |
|  | - | - | 475-484 | SEYNLKFDF | 473-482 | NLSEYNLKF | 242-251 | YLYSVYDQY |
|  | - | - | 146-155 | HEYYNTIKL | - | - | 458-467 | FQNYPIFLL |
| gudiv_427 | - | - | - | - | - | - | 76-85 | HKMFNLFSY |
| gudiv_442 | - | - | 124-133 | KEIISIINL | 39-48 | FITDEINSL | - | - |
|  | - | - | 211-220 | QQMIKYKKL | - | - | - | - |
| gudiv_457 | 138-147 | FSFVHTYPL | - | - | 7--16 | GLFRDSIPL | 114-123 | FQFEVEKFY |
|  | - | - | - | - | - | - | 136-145 | RLFSFVHTY |
|  | - | - | - | - | - | - | 138-147 | FSFVHTYPL |
|  | - | - | - | - | - | - | 161-170 | RKYTEVSQF |
| gudiv_458 | 152-161 | FSTTLKNHL | - | - | 10--19 | TLISSLVVL | - | - |
|  | 283-292 | IAYALNPTL | - | - | 159-168 | HLDGLYYQL | - | - |
|  | 287-296 | LNPTLNEVI | - | - | 192-201 | TLYFNKSQL | - | - |
|  | - | - | - | - | 367-376 | VLQKISNQL | - | - |
|  | - | - | - | - | 383-392 | NLTSDHFIL | - | - |
|  | - | - | - | - | 453-462 | NLEAAKINL | - | - |
|  | - | - | - | - | 460-469 | NLAKKQIDL | - | - |
| gudiv_499 | - | - | - | - | - | - | 36-45 | SKYEQIPTL |
| gudiv_517 | - | - | 43-52 | EQASLAYEL | - | - | 146-155 | ALYDLSGYF |
| gudiv_546 | - | - | 120-129 | SKYFKSFQL | 9--18 | MLLITSTSL | - | - |
|  | - | - | - | - | 33--42 | SIDPVTYQL | - | - |

**Additional file 3:** Sequence of the most representative CD8+ T lymphocyte epitopes and respective position in each UdLAMP predicted using NetBoLApan v1.0. Peptides with an 8-amino acid window of the BoLA-3 *00101, BoLA-4 *04101, BoLA-T2C, and BoLA-T5 alleles are represented.

**Conclusion**

| **UdLAMPs** | **BoLA-3 *00101** | | **BoLA-4 *04101** | | **BoLA-T2C** | | **BoLA-T5** | |
| --- | --- | --- | --- | --- | --- | --- | --- | --- |
|  | Position | Epitopes | Position | Epitopes | Position | Epitopes | Position | Epitopes |
| gudiv_560 | - | - | 94-103 | NEIKAVNQL | 152-161 | HLKTMVNTL | 322-331 | FMNQSSLLM |
|  | - | - | 286-295 | KESNHNNEL | 414-423 | QLFAGNTHL | 334-343 | EQYDLYKAY |
|  | - | - | - | - | - | - | 395-404 | LQANHILGF |
|  | - | - | - | - | - | - | 457-466 | IQRRFKTAF |
| gudiv_633 | - | - | 150-159 | NEYIIKKVL | 53-62 | TVVKQTQPL | 4--13 | IKFKIMASF |
|  | - | - | - | - | 229-238 | NTNEEFYSL | 99-108 | LSFGLNSLY |
|  | - | - | - | - | - | - | 410-419 | HSYYNNDYY |
| gudiv_635 | - | - | 144-153 | NEYIIKKVL | 56-65 | TVVKQTQPL | 93-102 | LSFKLDSSY |
|  | - | - | - | - | 221-230 | NTNEKFYSL | 431-440 | LEYNYIPRF |
| gudiv_663 | 335-344 | SADPLKNPL | 75-84 | QQYFRSGDL | 64-73 | ELAFQNQNL | 263-272 | ALYNFEFEY |
|  | - | - | 514-523 | DEKQQYANL | 206-215 | SVYAYATTM | 516-525 | KQQYANLAF |
|  | - | - | - | - | 283-292 | DLPQFGQPL | - | - |
|  | - | - | - | - | 391-400 | ELTSKNEAL | - | - |
|  | - | - | - | - | 546-555 | NLTKYVVDL | - | - |
| gudiv_680 | - | - | - | - | 8--17 | LMMVFLASL | 399-408 | GEYTITHVY |
|  | - | - | - | - | 208-217 | TLQVFINGL | - | - |
|  | - | - | - | - | 288-297 | DLIKTETEL | - | - |
|  | - | - | - | - | 295-304 | ELVKHNTEL | - | - |
| gudiv_681 | 30-39 | TNPTLKSEL | 130-139 | QEYKTKDLL | 211-220 | QLTLEVVNL | - | - |
| gudiv_759 | 102-111 | SSNDLINEL | 329-338 | KEENKPFSL | 316-325 | TLAELVMHL | 113-122 | GQFAAGLSY |
|  | - | - | 480-489 | EQKQKLYAL | 447-456 | ALANLGVNL | 119-128 | LSYNGDISF |
|  | - | - | - | - | 515-524 | DLSLANLKL | 183-192 | YKYINDISF |
|  | - | - | - | - | - | - | 248-257 | MRNFDYVAY |

-Absence of epitopes
